# Metabolic shifts in tryptophan pathways during acute pancreatitis infections

**DOI:** 10.1172/jci.insight.186745

**Published:** 2025-03-10

**Authors:** Daosheng Wang, Silei Sun, Qianli Zhao, Bing Zhao, Li Ma, Tongxuan Su, Lili Xu, Menglu Gui, Dan Xu, Wei Chen, Yu Zeng, Yining Shen, Yiyue Liu, Cen Jiang, Qi Ni, Yingchao Cui, Yide Lu, Qiuya Lu, Danfeng Dong, Yibing Peng, Enqiang Mao

**Affiliations:** 1 Department of Emergency,; 2 Department of Laboratory Medicine, and; 3 Department of Critical Care Medicine, Ruijin Hospital, Shanghai Jiao Tong University School of Medicine, Shanghai, China.; 4 Faculty of Medical Laboratory Science, College of Health Science and Technology, Shanghai Jiao Tong University School of Medicine, Shanghai, China.

**Keywords:** Gastroenterology, Amino acid metabolism, Bacterial infections, Diagnostics

## Abstract

Infectious complications (ICs) in acute pancreatitis (AP) are primarily driven by intestinal bacterial translocation, significantly increasing mortality and hospital stays. Despite this, the role of the gut microenvironment, particularly its metabolic aspects, in AP remains poorly understood. In this study, we investigated a cohort of patients with AP, and conducted supplemental murine studies, to explore the relationship between the gut metabolome and the development of ICs. Metabolomic analysis revealed that disruptions in gut tryptophan metabolism — especially reductions in serotonin and indole pathways — are key features associated with IC occurrence. Additionally, elevated plasma levels of tryptophan metabolites within the kynurenine pathway were identified as valuable predictive biomarkers for ICs. Mechanistic studies in murine models demonstrated that an impaired intestinal Th17 response, modulated by these tryptophan metabolites, plays a critical role in IC development. Serotonin supplementation enhanced Th17 responses, reducing IC incidence, while administration of kynurenic acid, a kynurenine metabolite, exacerbated pancreatic infections, potentially through immunosuppressive effects. These findings highlight the pivotal role of tryptophan metabolites in AP pathogenesis, emphasizing their potential as both predictive markers and therapeutic targets in IC management.

## Abstract 2

Dysregulated tryptophan metabolism indicates the development of infectious complications in acute pancreatitis, offering new insights for early diagnosis and therapeutic targets.

## Graphical abstract

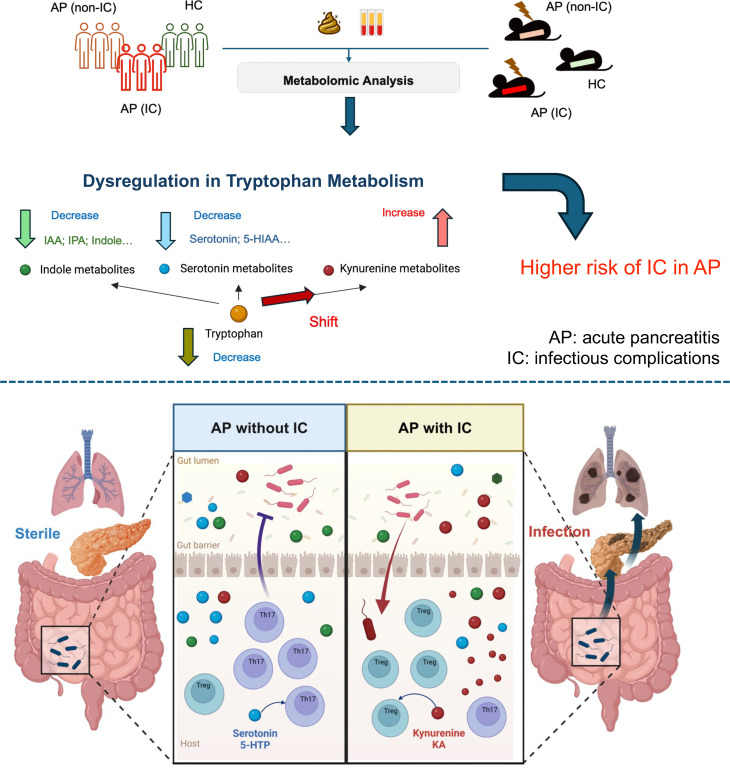

## Introduction

Acute pancreatitis (AP) results from the abnormal activation of pancreatic enzymes, leading to self-digestion of pancreatic tissue and sudden abdominal pain (1). Common causes include gallstones, hyperlipidemia, and alcohol consumption (1). The revised Atlanta classification system categorizes severity as mild, moderate, or severe, with severe AP based on organ failure within the initial 48 hours (1). For severe acute pancreatitis (SAP) cases, infectious complications (ICs), particularly infected pancreatic necrosis (IPN), can result in rapid disease progression, leading to significantly longer hospital stays and increased mortality rates of 20%–40% (2–5). This highlights the importance of early prediction of ICs to guide prompt and effective clinical decision-making for patients with SAP. However, the search for sensitive and reliable biomarkers remains ongoing.

Intestinal bacterial translocation, a process where viable luminal microbes breach the compromised gut barrier and trigger infections in distant organs, is widely recognized as the primary instigator of ICs in AP (6, 7). With the hypothesis that indicators of IC occurrence may manifest in the gut lumen during the initial phases of AP, our current study focused on the alterations in the gut of AP. Although previous research has shed light on the role of the gut contents in manipulating AP progression (8–10), most studies concentrated on early inflammation, leaving their roles in bacterial translocation largely unexplored. Furthermore, the gut metabolites originating from diet, host metabolism, and microbiota metabolism play crucial roles in preserving gut barriers and antimicrobial defenses during critical illness (11–13), yet this aspect is less studied compared with the microbiome in AP. Therefore, our study aims to investigate the alterations in gut metabolic profiles in AP and their potential association with the development of ICs. The insights gained from this research provide valuable perspectives on the mechanisms underlying intestinal bacterial translocation in AP and may contribute to identifying biomarkers to predict ICs in this condition.

## Results

### Study population.

To investigate the relationship between metabolic features and the occurrence of ICs in AP, we recruited a cohort of 92 patients with AP from the emergency intensive care units (ICUs) at Ruijin Hospital, Shanghai, China, during our clinical observation period. The clinical and demographic characteristics of the patients can be found in Supplemental Table 1; supplemental material available online with this article; https://doi.org/10.1172/jci.insight.186745DS1 The most common causes of AP in this cohort were bile and hyperlipidemia, with 49 cases categorized as SAP. During the course of the disease, 43 patients developed ICs. The median interval between infection diagnosis and hospital admission was 5 days. The primary types of infections included pulmonary (*n* = 35), urinary (*n* = 14), and abdominal (*n* = 14). The primary pathogens included *Acinetobacter baumannii* (*n* = 16) and *Klebsiella pneumoniae* (*n* = 12). Additionally, 12 patients developed IPN, and 19 patients developed infections in more than 2 sites. Importantly, those who developed infections or specifically IPN experienced significantly longer hospital and ICU stays, along with higher Acute Physiology and Chronic Health Evaluation II (APACHE II) scores, Bedside Index for Severity in Acute Pancreatitis (BISAP) scores, and serum procalcitonin (PCT) levels (Supplemental Tables 2 and 3). Patients with infections in multiple sites had even longer hospital and ICU stays compared with those with infections in a single site (Supplemental Table 4). These findings emphasized the more severe disease progression in patients with AP who developed ICs.

### Dysregulation of gut tryptophan metabolism is associated with AP disease progression.

**Figure 1:**
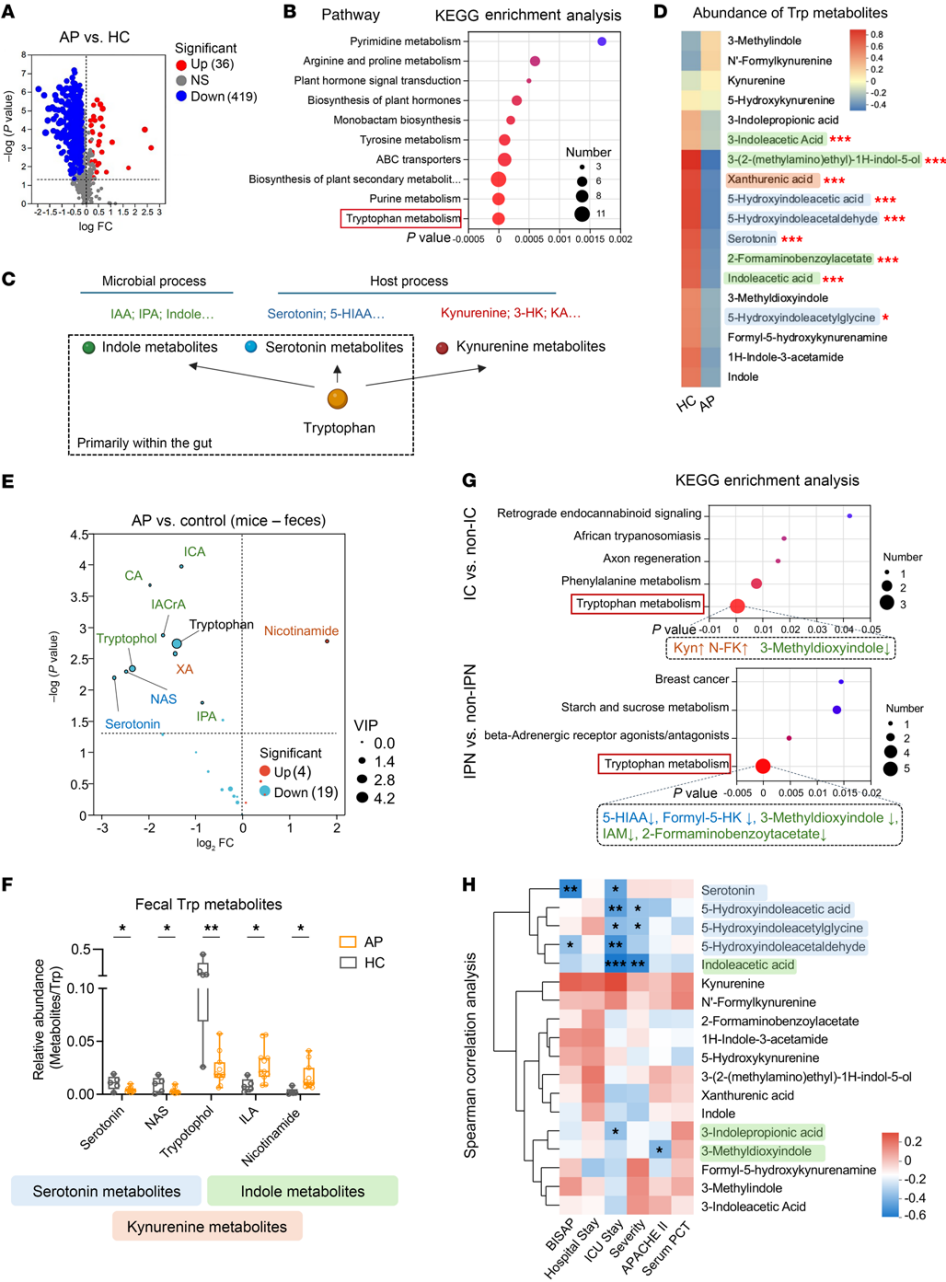
Alterations in fecal tryptophan metabolism in human AP patients and mouse models. (**A**) Volcano plot of differential metabolites between patients with AP (*n* = 33) and healthy controls (HCs; *n* = 19). Depleted (blue) and enriched (red) metabolites are highlighted based on *P* < 0.05 and variable importance in projection (VIP) > 1. (**B**) Bubble diagram showing Kyoto Encyclopedia of Genes and Genomes (KEGG) pathway enrichment analysis of differential gut metabolites between patients with AP (*n* = 33) and HCs (*n* = 19). Bubble size indicates the number of enriched metabolites, while the color gradient reflects enrichment significance. (**C**) Schematic of host and microbial tryptophan metabolism pathways. (**D**) Heatmap showing the relative abundance of tryptophan (Trp) metabolites across human study groups. (**E**) Volcano plot of differential fecal metabolites between TCA-induced AP mice (*n* = 11) and HC mice (*n* = 5). Depleted (blue) and enriched (red) metabolites were identified using a significance threshold of *P* < 0.05 (Student’s *t* test) and VIP > 0.1. (**F**) Relative abundance (Metabolite/Trp) of significant Trp metabolites in fecal samples from AP (*n* = 11) and HC mice (*n* = 5). (**G**) Bubble diagram of KEGG enrichment analysis of differential gut metabolites between AP patients with and without IC (IC, *n* = 16; non-IC, *n* = 17) and between AP patients with or without IPN (IPN, *n* = 5; non-IPN, *n* = 28). Enriched and depleted Trp metabolites are indicated by upward or downward arrows, respectively. (**H**) Correlation heatmap showing the relationships between clinical severity parameters and fecal Trp metabolites in patients with AP (*n* = 33). Box plots display medians and quartiles. Statistical analyses: Mann-Whitney test (**A** and **D**), Fisher’s exact test (**B** and **G**), Student’s *t* test (**E** and **F**), and Spearman’s correlation (**H**). *P < 0.05, **P < 0.01, ***P < 0.001.

We initially employed untargeted liquid chromatography–tandem mass spectrometry (LC-MS/MS) metabolomics to identify potentially significant intestinal metabolic pathways in AP using a randomly selected discovery group. Compared with healthy controls (HCs), we observed a significant reduction in the levels of the majority of gut metabolites in AP (Figure 1A). These altered metabolites were primarily associated with tryptophan metabolism, purine metabolism, and the biosynthesis of plant secondary metabolites, with tryptophan metabolism displaying the most significant *P* value (Figure 1B). The tryptophan metabolism involves 3 pathways: the kynurenine (Kyn) and serotonin (Ser) pathways, which are metabolized by the host, and the indole (Ind) pathways, primarily managed by the gut microbiota (Figure 1C) (14). Notably, we found that the reduced intestinal tryptophan metabolites in AP were mainly involved in the Ser and Ind pathways (Figure 1D). To validate the dysregulation of tryptophan metabolism in human AP, we utilized a sodium taurocholate acid–induced (TCA-induced) AP mouse model and performed a targeted metabolomic analysis of mouse stool samples, accurately quantifying 33 common tryptophan metabolites. Our results revealed that AP mice exhibited significant depletions in tryptophan as well as several Ser and Ind metabolites, including Ser, *N*-acetyl-serotonin (NAS), tryptophol, and 3-indoleproponic acid (IPA), compared with HC mice (Figure 1E). Due to the depletion of tryptophan substrate, we also utilized the relative abundance of these metabolites, calculated as the ratio of metabolite concentration to tryptophan concentration, as an indicator for pathway assessment. Through this analysis, we noted a decrease in the relative abundance of 2 Ser metabolites, Ser and NAS, in AP mice as well (Figure 1F). Conversely, both the concentration and relative abundance of nicotinamide, a Kyn metabolite, increased in AP mice compared with that in HC mice (Figure 1, E and F).

Next, we investigated the association between the gut metabolome and the future progression of AP in patients. Intriguingly, comparison of patients with AP who developed ICs during the disease course (AP-IC group) with those who did not (AP-non-IC group) revealed that the differential metabolites were predominantly enriched in the tryptophan metabolism pathway (Figure 1G). This pathway was also identified as the metabolic pathway most significantly associated with the occurrence of IPN (Figure 1G). Specifically, AP patients with ICs, or IPN specifically, exhibited marked reductions in the Ser and Ind pathways, accompanied by a potential augmentation of the Kyn pathway, compared with AP patients without ICs or IPN (Figure 1G). Furthermore, we found significant negative correlations among the abundance of Ser and Ind metabolites and disease outcomes, including ICU stay duration and serum PCT levels (Figure 1H). This observation suggests potential alleviating impacts of gut Ser and Ind metabolites in the progression of AP. In summary, our findings underscored the presence of dysregulated tryptophan metabolism in the intestine during AP that was characterized by diminished Ser and Ind pathway activities and closely linked to disease progression.

### The AP gut microbiota and its association with ICs.

We also assessed the microbial alterations in AP using 16S rRNA sequencing. Evaluation of the gut microbiome health index (GMHI) (15) revealed significantly lower values in patients with AP upon admission and AP mice compared with those in the corresponding HC groups, indicating dysregulation in the gut microbiota of AP (Supplemental Figure 1, A and B). Specifically, we observed increased abundances of opportunistic pathogens, including *Enterococcaceae* and *Enterobacteriaceae*, with decreased abundance of *Lachnospiraceae*, specifically *Blautia* spp., in both patients and mice with AP (Supplemental Figure 1, C and D). We further evaluated the association between the gut microbiota and the development of ICs, which revealed that patients with ICs exhibited a significantly lower GMHI and a notably higher abundance of *Enterobacteriaceae* (Supplemental Figure 1, E and F). *Enterobacteriaceae* was also more abundant in patients with IPN than those with AP without IPN (Supplemental Figure 1G). Furthermore, we found that higher levels of *Enterobacteriaceae* in AP were significantly associated with longer hospital stays (Supplemental Figure 1H). Overall, these findings indicate that the initial microbial composition upon admission, particularly the heightened presence of *Enterobacteriaceae*, can also serve as a potential predictor for subsequent secondary infections in the setting of AP.

### Plasma Kyn metabolite levels predict future IC occurrence in AP.

In the process of tryptophan metabolism, the Ser and Ind pathways mainly occur within the gut, whereas the Kyn pathway takes place in various locations throughout the host (14, 16). To capture the full tryptophan metabolic landscape, we then conducted an analysis of the plasma untargeted metabolomic data from the discovery group of patients with AP. Among the annotated tryptophan metabolites, kynurenic acid (KA), a vital metabolite of the Kyn pathway, significantly increased in the plasma of AP compared with HCs (Supplemental Figure 2A). Moreover, KA level was much higher in AP patients with IPN and showed significant correlations with the length of stay in the ICU (Supplemental Figure 2, B and C).

**Figure 2:**
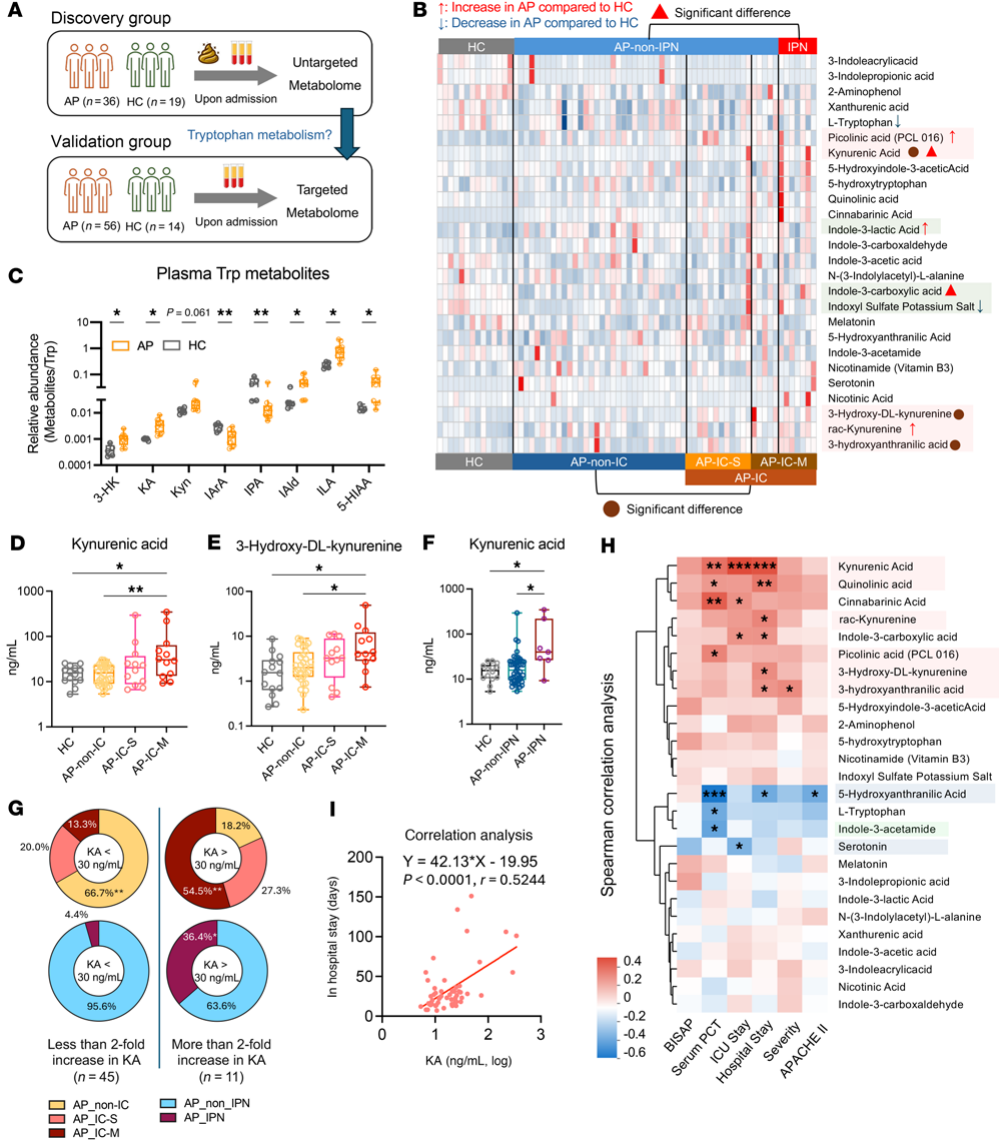
Plasma tryptophan profiling in relation to ICs in AP. (**A**) Flow chart outlining the study design for identifying meaningful tryptophan metabolites in patients with AP. (**B**) Heatmap showing the abundance of tryptophan metabolites in the validation group. Significant differences were evaluated between AP (*n* = 56) and HC (*n* = 14), AP-non-IC (*n* = 32) and IC (*n* = 24), and AP-non-IPN (*n* = 49) and IPN (*n* = 7). Differential metabolites were identified as *P* < 0.05 with VIP > 0.1 and are labeled. AP-IC-M, AP with multisite IC; AP-IC-S, AP with single-site IC. (**C**) Relative abundance (Metabolite/Trp) of significant tryptophan metabolites in plasma from AP-induced mice (*n* = 11) and HC mice (*n* = 5). (**D**–**F**) Plasma levels of kynurenic acid (KA) and 3-hydroxy-dl-kynurenine (3-HK) in AP subgroups. (**D**) KA levels in AP-non-IC (*n* = 32), AP-IC-S (*n* = 12), AP-IC-M (*n* = 12), and HC (*n* = 14). (**E**) 3-HK levels in the same groups. (**F**) KA levels in AP-IPN (*n* = 7), AP-non-IPN (*n* = 48), and HC (*n* = 14). (**G**) Distribution of multisite IC, single-site IC, and non-IC, as well as IPN and non-IPN, among AP patients with KA levels above or below 30 ng/mL (2-fold HC levels). (**H**) Correlation heatmap of clinical severity parameters and plasma tryptophan metabolites in patients with AP (*n* = 55). (**I**) Correlation between plasma KA levels (log, ng/mL) and total hospital stay (*n* = 55). One patient who was discharged early was excluded from **H** and **I**. Box plots display medians and quartiles. Statistical analyses: Mann-Whitney test (**B**); Student’s *t* test (**C**); Kruskal-Wallis with 2-stage linear step-up procedure of Benjamini, Krieger, and Yekutieli (**D**–**F**); χ^2^/Fisher’s exact test (**G**); or Spearman’s (**H**) or Pearson’s (**I**) correlation. *P < 0.05, **P < 0.01, ***P < 0.001. See also Supplemental Figures 3 and 4.

Given the precision limitations of untargeted metabolomic tools, we proceeded to perform a targeted analysis of plasma tryptophan metabolites in a validation group comprising 56 patients with AP (Figure 2A). Compared with the HC group, we found significant depletion of plasma tryptophan levels in AP, along with elevated Kyn levels and an increased kynurenine/tryptophan (Kyn/Trp) ratio (Figure 2B and Supplemental Figure 3A). Notably, this elevation in Kyn levels and the Kyn/Trp ratio was even more pronounced in the SAP group (Supplemental Figure 3, B and C). In the AP mouse model, AP resulted in decreased plasma tryptophan levels but an increased relative abundance of several Kyn metabolites, including 3-hydroxy-dl-kynurenine (3-HK) and KA (Figure 2C and Supplemental Figure 3D). These findings suggest that alterations in tryptophan metabolism may extend beyond the gut environment and manifest in the circulating plasma during AP. Integrating those metabolomic analyses, we conclude that the downregulation of the Ser and Ind pathways in the intestine, combined with the upregulation of the Kyn pathway in the plasma, represents a key metabolic feature of tryptophan metabolism in AP.

We next assessed the associations between plasma metabolites and AP progression. We observed that patients with AP who developed ICs had significantly elevated levels of Kyn metabolites, specifically 3-HK and KA, upon admission (Figure 2B). This trend was particularly pronounced in patients with multisite infections (Figure 2, D and E). Patients with IPN showed significantly higher levels of KA compared with other patients with AP (Figure 2F). We also compared each specific infection type — pulmonary, urinary, and abdominal infections — with the non-IC group and observed that increases in Kyn metabolites, including KA, quinolinic acid, and 3-HK, were consistently present across these infection types (Supplemental Figure 4, A–C). When stratifying patients with AP into 2 groups based on whether their metabolite levels exceeded a 2-fold increase relative to HCs, we found that patients with markedly elevated levels of KA and 3-HK were significantly more likely to develop ICs, including multisite infections (Figure 2G and Supplemental Figure 4D). Additionally, elevated KA levels were associated with an increased likelihood of developing IPN (Figure 2G). In summary, these findings suggest that elevated Kyn metabolites, such as KA and 3-HK, may serve as indicators of a heightened risk for developing ICs in AP.

**Figure 3:**
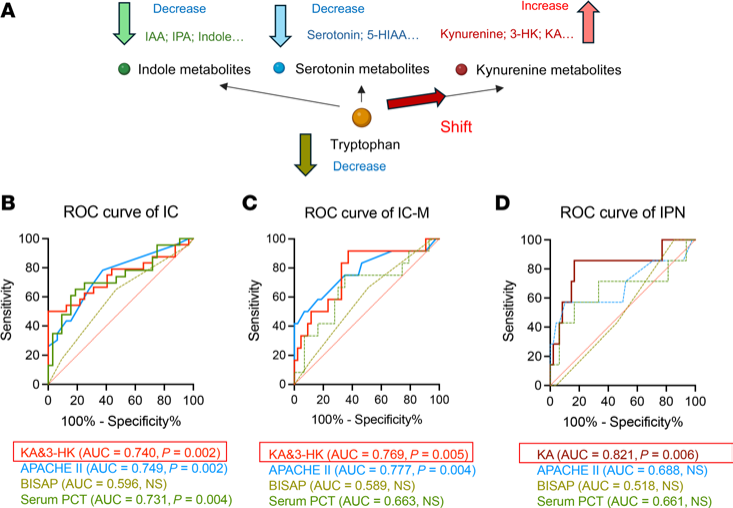
Plasma kynurenine metabolites predict the development of ICs in AP. (**A**) Imbalance in tryptophan metabolism in AP linked to the risk of ICs. In AP patients with ICs, tryptophan metabolism shifts predominantly toward the kynurenine pathway, resulting in elevated levels of metabolites such as kynurenine, 3-HK, and KA. Concurrently, the intestinal indole and serotonin pathways are diminished, leading to a depletion of metabolites like IAA, IPA, serotonin, and 5-HIAA in the intestine. (**B**–**D**) Receiver operating characteristic (ROC) analysis of ICs (**B**), multisite IC (**C**), and IPN (**D**) outcomes using plasma tryptophan metabolites (3-HK & KA or KA alone), APACHE II scores, serum PCT, and BISAP scores among patients with AP. AUC, area under the curve; IAA, indole-3-acetic acid.

We then conducted a correlation analysis, which revealed significant positive associations between specific Kyn metabolites, particularly KA, and prolonged hospital and ICU stays, while Ser metabolites exhibited weak negative correlations (Figure 2H). On average, a 0.1 log increase in KA was associated with an additional 4.2 days of hospitalization (Figure 2I). Integrating these findings with fecal metabolomic data further reinforced the strong association between altered tryptophan metabolism and the progression of AP. The distinct distribution and correlation patterns of Kyn and Ser metabolites highlighted their contributions to the development of ICs in AP (Figure 3A).

Since all patient samples were collected upon admission, the observed correlation between elevated circulatory Kyn metabolite levels and ICs suggests their potential value in predicting infectious outcomes in AP. Utilizing the 10-fold cross-validation method, we identified potential metabolic biomarkers for predicting ICs. The combined index, comprising levels of KA and 3-HK, demonstrated good performance in predicting IC occurrence in AP, with an area under the curve (AUC) of 0.740, which was comparable to the APACHE II score and serum PCT level (Figure 3B). Additionally, this combined index exhibited strong predictive performance for multisite ICs in AP, with an AUC of 0.769, outperforming the serum PCT level (Figure 3C). Importantly, plasma KA demonstrated excellent predictive capability for IPN occurrence, with an AUC of 0.821, thereby surpassing both the APACHE II score and serum PCT level (Figure 3D). Overall, our findings present valuable metabolic indicators for assessing the risk of future development of ICs in AP.

### Impaired intestinal Th17 cell response drives ICs in AP.

Next, we aimed to explore the underlying mechanisms that link tryptophan metabolism to the development of ICs in AP. One of the key factors contributing to ICs in AP is intestinal bacterial translocation, which involves the migration of live bacteria across the intestinal barrier to other organs or the circulatory system, primarily occurring in the small intestine (6, 7). Our findings, showing significantly reduced positive staining areas for the tight junction proteins Zo-1 and Occludin in the small intestines of AP mice compared with HC, supported the likelihood of bacterial translocation (Supplemental Figure 5, A–C). Previous research has shown that tryptophan metabolism influences both epithelial integrity and antimicrobial defense in the intestine (6, 14, 17, 18), which possibly affect the process of intestinal bacterial translocation that leads to ICs. To further investigate the effects of tryptophan metabolites, we initially examined essential intestinal processes related to the development of ICs in AP mice.

**Figure 4:**
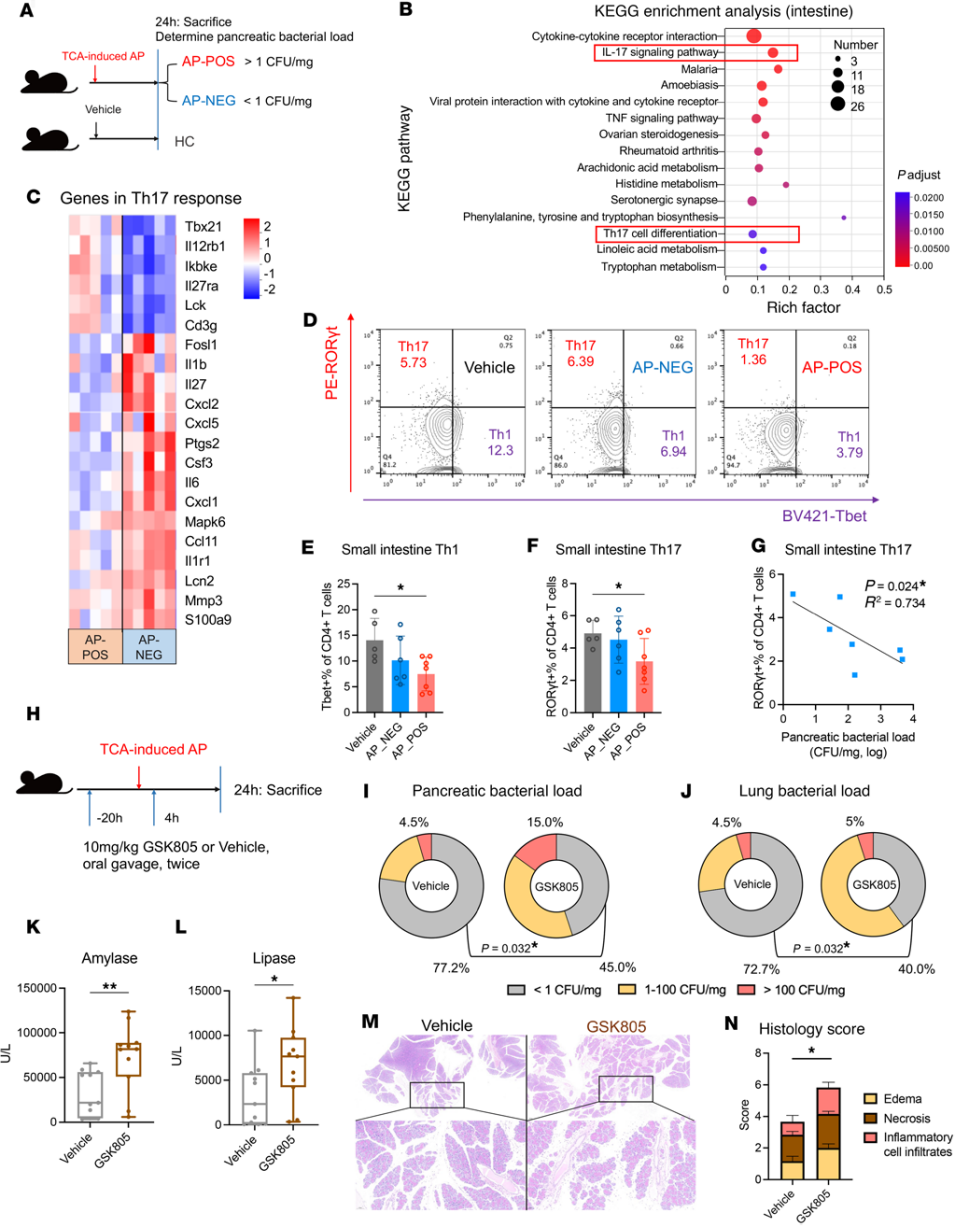
Impaired intestinal Th17 cell responses facilitate infections in AP mice. (**A**) Schematic diagram of comparison groups. Pancreatic bacterial loads >1 CFU/mg were classified as AP-POS and <1 CFU/mg as AP-NEG, and vehicle group mice were classified as HCs. (**B**) KEGG enrichment analysis of intestinal gene sets associated with AP infection. Pathways associated with Th17 response are highlighted in red box. (**C**) Heatmap showing the differential expression levels of genes associated with the intestinal Th17 response in the AP-POS and AP-NEG groups. (**D**–**F**) Flow cytometry assessment of Th1 (T-bet^+^RORγt^–^) and Th17 (T-bet^–^RORγt^+^) populations among CD4^+^ T cells in the small intestinal lamina propria of the vehicle (*n* = 5), AP-NEG (*n* = 6), and AP-POS (*n* = 7) groups. BV, brilliant violet; PE, phycoerythrin; ROR, retinoic acid–related orphan receptor. (**G**) Correlation between the proportion of Th17 cells in the small intestine and the pancreatic bacterial load among AP group mice. (**H**) Schematic diagram illustrating 2 administrations of GSK805 (10 mg/kg) by oral gavage: once 20 hours before AP induction and again 4 hours after induction. (**I** and **J**) Comparison of bacterial loads in the pancreas (**I**) and lungs (**J**) between GSK805-administered AP mice (*n* = 20) and controls (*n* = 22). (**K** and **L**) Comparison of plasma amylase (**K**) and lipase (**L**) levels between GSK805-treated and vehicle-treated mice (*n* = 11 per group). (**M** and **N**) Pancreatic histology in vehicle- and GSK805-treated groups (*n* = 6 each). (**M**) H&E staining at 5× and 20× original magnifications. (**N**) Pathological scoring of the groups. Error bars represent the mean ± SD. Box plots depict the median and quartiles of each group. Statistical analyses: 1-way ANOVA with Tukey’s test (**E** and **F**), Student’s *t* test (**N**), Fisher’s test (**B**, **H**, and **I**), Mann-Whitney test (**J** and **K**), or Spearman’s correlation test (**G**); *P < 0.05, **P < 0.01. See also Supplemental Figures 6 and 7.

Through RNA sequencing to elucidate gene expression profiles, we discovered that genes with upregulated expression in small intestinal tissues of AP mice were predominantly enriched in cytokine effect pathways, while those with downregulated expression were primarily associated with amino acid absorption and metabolism, as well as T cell differentiation (Supplemental Figure 6, A–C). Subsequently, based on pancreatic bacterial detection results, we divided AP mice into infection (AP-POS) and noninfection (AP-NEG) groups (Figure 4A), which facilitated the identification of 589 differentially expressed genes associated with pancreatic infection in AP (Supplemental Figure 6D). Notably, these genes were enriched in the IL-17 signaling pathway and Th17 differentiation (Figure 4B), which are essential for mucosal antimicrobial defense (19), thereby emphasizing the strong association between Th17 response and pancreatic infection. Specifically, AP-POS mice exhibited lower expression of key cytokine genes (*Il6* and *Il1b*) involved in regulating Th17 differentiation (20), as well as peptides (*Lcn2* and *S100a9*) that respond to IL-17 to combat invading pathogens (19) (Figure 4C). To validate these transcriptional changes, we evaluated the immune cell populations within the lamina propria of the small intestine. Compared with vehicle control mice, AP-POS mice exhibited significant decreases in Th1 and Th17 populations (Figure 4, D–F, and Supplemental Figure 7A). Additionally, mice with a pancreatic bacterial load exceeding 100 CFU/mg exhibited the most pronounced reduction in Th17 cells, whereas Th1 and regulatory T cells (Tregs) were less affected (Supplemental Figure 7, A–C). AP-POS mice also showed decreased expression of *Il17a* in the lamina propria, while *Ifng* expression levels remained unchanged (Supplemental Figure 7D). Furthermore, unlike Th1, the Th17 population showed a significant inverse correlation with pancreatic bacterial load, suggesting that a weakened Th17 response may contribute to the promotion of infections (Figure 4G and Supplemental Figure 7E). In addition, we compared the extent of barrier disruption across groups. Both AP-NEG and AP-POS groups showed decreased expression levels of intestinal tight junction proteins and elevated plasma D-lactic acid (D-lac) — a marker of gut injury — compared with the HC group, indicating gut barrier disruption in AP (Supplemental Figure 7, F and G). However, the comparable levels between AP-NEG and AP-POS suggest that gut integrity disruption may not play as central a role as the Th17 response in this context.

To verify the role of the Th17 response in IC development, we administered the RORγt inhibitor (GSK805) to AP mice, which successfully decreased intestinal IL-17A expression (Figure 4H and Supplemental Figure 7H). Notably, there were significantly higher levels of positive bacterial detection in the pancreas and lungs of AP mice treated with GSK805 compared with those treated with the vehicle control (Figure 4, I and J). Furthermore, pancreatic injury, as indicated by plasma amylase and lipase levels as well as histopathological changes, was more severe under Th17 response inhibition ((Figure 4, K–N). In summary, these findings underscored the crucial importance of inadequate Th17 response in driving the occurrence of ICs in the AP intestine.

### Supplementation with 5-hydroxytryptophan modulates intestinal Th17 response and improves AP.

**Figure 5:**
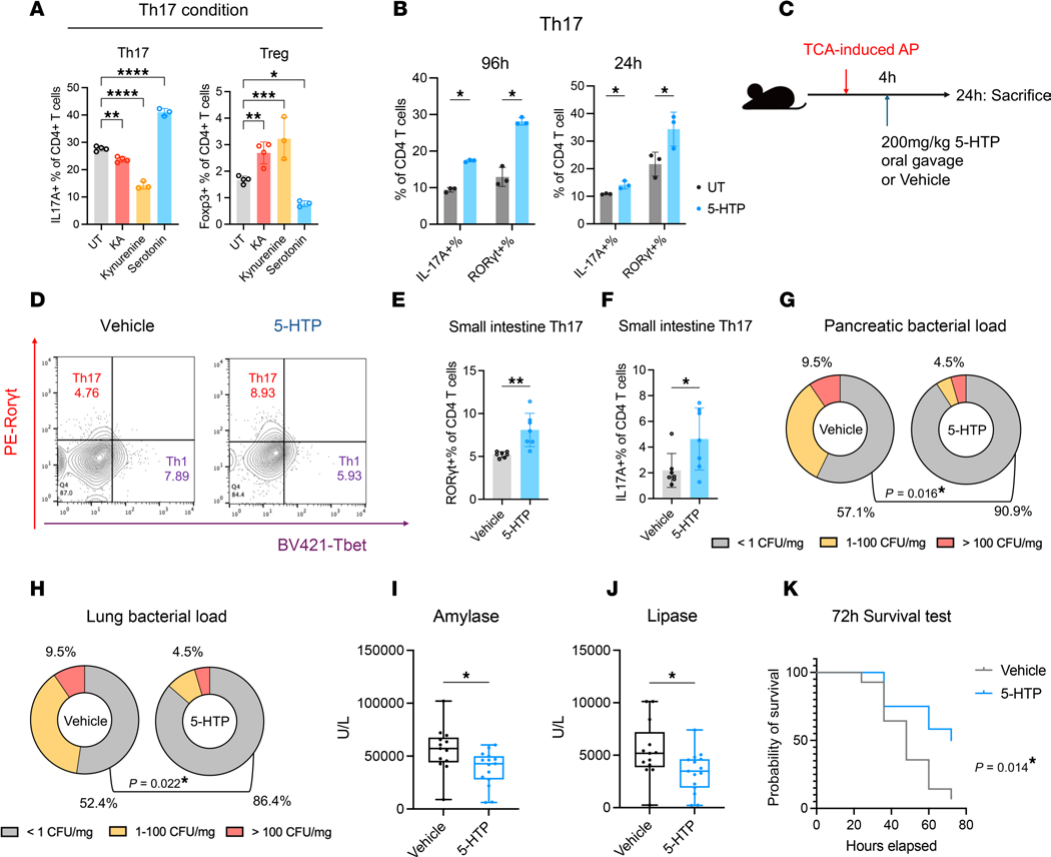
Administration of 5-hydroxytryptophan enhances Th17 responses and alleviates AP. (**A**) Effects of 100 μM KA, kynurenine, or serotonin on the polarization of murine primary spleen naive CD4^+^ T cells toward Th17 compared with an untreated (UT) group (*n* = 3–4 per group). (**B**) Effect of long-term (96 hours) and short-term (24 hours) stimulation with 100 μM 5-HTP on the polarization of murine primary spleen naive CD4^+^ T cells toward Th17 (*n* = 3 per group). (**C**) Schematic diagram illustrating the administration of 5-HTP (200 mg/kg) via oral gavage at 4 hours after AP induction with TCA. (**D**–**F**) Flow cytometry analysis of the Th17 population in the small intestinal lamina propria of AP mice (*n* = 6–7 per group), with and without 5-HTP supplementation, using PE-labeled RORγt and BV421-labeled T-bet. The Th17 population was identified using 2 markers: RORγt^+^CD4^+^% (**E**) and IL-17A^+^CD4^+^% (**F**). (**G** and **H**) Comparisons of bacterial loads in the pancreas (**G**) and lung (**H**) between 5-HTP– and vehicle-treated AP mice (*n* = 21 per group). (**I** and **J**) Comparisons of plasma amylase (**I**) and lipase (**J**) levels between 5-HTP–treated (*n* = 15) and vehicle-treated (*n* = 13) AP mice. (**K**) Survival curves for 5-HTP–treated (*n* = 12) and vehicle-treated (*n* = 14) AP mice. Error bars represent the mean ± SD. Box plots depict the median and quartiles of each group. Statistical analyses: Student’s *t* test (**A**, **B**, **E**, **F**, **I**, and **J**), Fisher’s test (**G** and **H**), and log-rank (Mantel-Cox) test (**K**). *P < 0.05, **P < 0.01, ***P < 0.001. See also Supplemental Figures 8 and 9.5-HTP, 5-hydroxytryptophan.

Tryptophan metabolites play important roles in maintaining the intestinal immune barriers (14). Given the essential role of Th17 cells in immune defense against infections, we postulated that these particular tryptophan metabolites may influence the infections by affecting intestinal Th17 cells. To test this hypothesis, we exposed primary spleen CD4^+^ T cells from mice to various metabolites at a concentration of 100 μM under conditions optimized for Th17 cell differentiation. Our observations revealed that Ser significantly increased the production of IL-17A^+^ cells, while Kyn and KA had the opposite effect (Figure 5A). Notably, exposure to Kyn or KA also simultaneously increased the percentage of Foxp3^+^ Tregs under Th17 differentiation conditions (Figure 5A). When applying a lower concentration of 20 μM, the enhancement of Th17 cell production by Ser remained evident, while Kyn continued to show a clear boost in Treg percentages (Supplemental Figure 8). The varied impacts of different tryptophan metabolites on Th17 cells may explain their diverse correlations with clinical ICs in AP.

Considering the enhancing effect of Ser on Th17 cells and its negative association with AP progression, we hypothesized that supplementation with Ser could avoid infections in AP. In practical applications, Ser is typically supplemented in the form of the Ser precursor 5-HTP. Using in vitro assessments, we observed that both long-term (96 hours) and short-term (24 hours) exposure to 100 μM 5-HTP increased IL-17A and RORγt levels in Th17 cells (Figure 5B). This effect was similarly observed at the lower concentration of 20 μM (Supplemental Figure 8). Next, we used oral gavage to administer 5-HTP to mice at 4 hours after AP induction (Figure 5C). Administration of 5-HTP led to a significant increase in the levels of small intestinal Th17 cells, as indicated by the proportion of RORγt^+^ and IL-17A^+^ cells, while the levels of Th1 cells and Tregs remained unchanged (Figure 5, D–F, and Supplemental Figure 9, A–C). Additionally, the expression levels of 2 downstream antimicrobial peptides associated with Th17 response (Defa5 and Reg3g) and 2 barrier molecules (Tjp1 and Muc2) were all augmented following 5-HTP supplementation (Supplemental Figure 9, D–G). These results suggested that 5-HTP has the potential to improve mucosal immune defense. Furthermore, 5-HTP supplementation of AP mice remarkably reduced the occurrence of pancreatic and lung infections (Figure 5, G and H) and alleviated the pancreatic injury, as evidenced by decreased levels of amylase and lipase (Figure 5, I and J). Moreover, 5-HTP administration improved the survival rate of AP mice over a 72-hour period (Figure 5K). Collectively, these findings suggested that 5-HTP supplementation could be a valuable therapeutic strategy to mitigate the occurrence of ICs in AP.

### KA might affect AP progression through immunosuppression.

**Figure 6:**
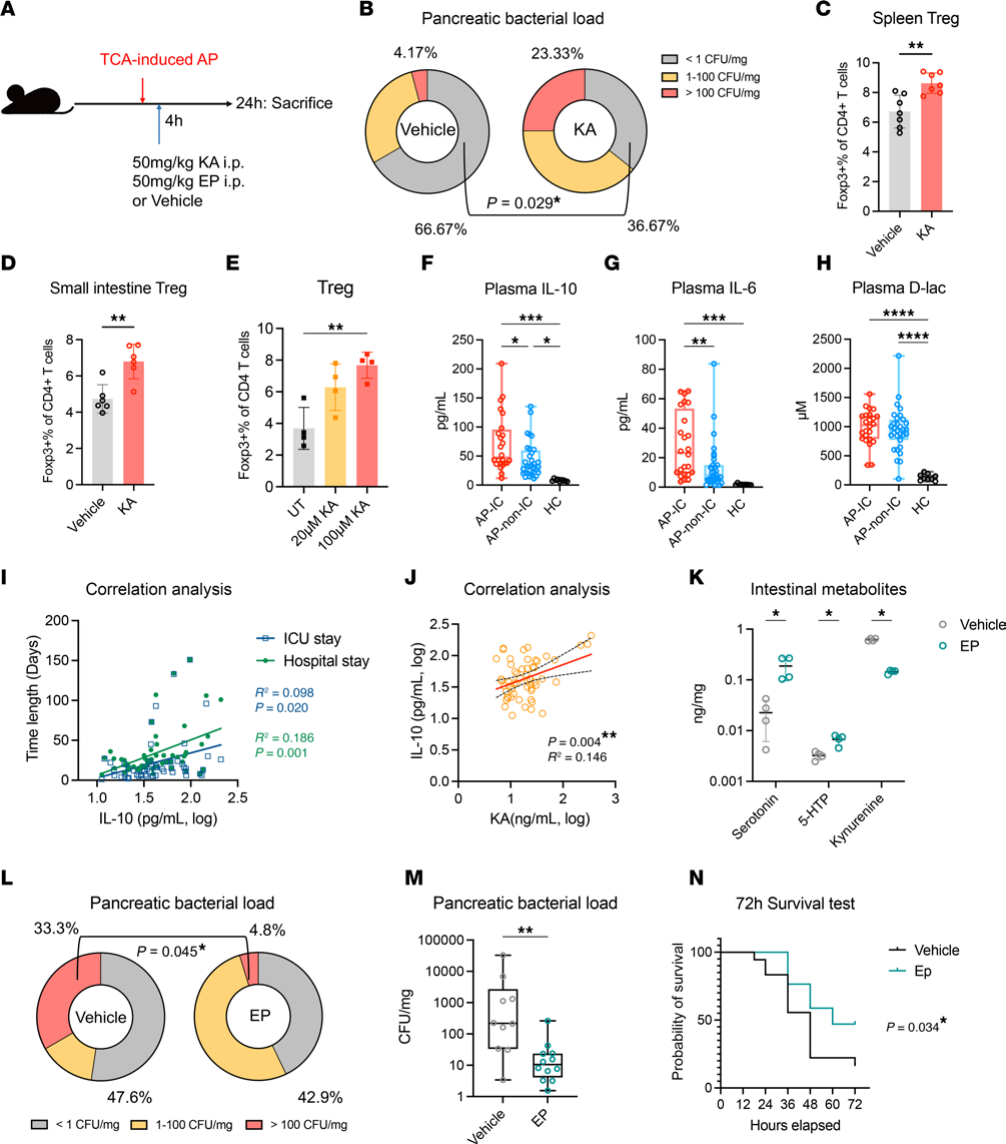
KA administration might aggravate AP through immunosuppression. (**A**) Schematic diagram illustrating intraperitoneal (i.p.) injection of 50 mg/kg KA or epacadostat (EP) at 4 hours after AP induction with TCA. (**B**) Comparisons of bacterial loads in the pancreas between KA- and vehicle-treated AP mice; vehicle (*n* = 24), KA (*n* = 28). (**C** and **D**) Evaluation of proportions of Tregs in the spleen (**C**) and small intestinal lamina propria (**D**) of AP mice, with and without KA administration (*n* = 6–7 per group). (**E**) Effect of 20 μM and 100 μM KA or no treatment (UT) on the polarization of murine primary spleen naive CD4^+^ T cells toward Tregs (*n* = 4 per group). (**F**–**H**) Plasma levels of IL-10, IL-6, and d-lactic acid (D-lac) among the groups of AP patients with (AP-IC; *n* = 24) and without (AP-non-IC; *n* = 31) ICs and HCs (*n* = 9). (**I**) Correlation between IL-10 level and lengths of ICU stay and hospital stay among patients with AP (*n* = 55). (**J**) Correlation between KA concentration and plasma IL-10 level among patients with AP (*n* = 55). (**K**–**M**) Effects of vehicle or EP treatment of AP mice on ileal tryptophan metabolites (*n* = 4 per group, **K**) and pancreatic bacterial load (*n* = 21 per group, **M**). (**N**) Survival curves for vehicle-treated (*n* = 18) and EP-treated (*n* = 17) AP mice. Error bars represent the mean ± standard deviation. Box plots depict the median and quartiles of each group. Data analyzed using Student’s *t* test (**C**, **D**, and **K**), Fisher’s test (**B** and **L**), 1-way ANOVA with Tukey’s test (**E**–**H**), Pearson’s correlation (**I** and **J**), Mann-Whitney test (**M**), and log-rank (Mantel-Cox) test (**N**). *P < 0.05, **P < 0.01, ***P < 0.001. See also Supplemental Figures 10–12.

We then assessed the impact of plasma Kyn metabolites on the development of ICs in AP. Given its strong correlation with ICs in our previous analysis, KA was specifically evaluated in an AP mouse model (Figure 6A). Although i.p. administration of KA did not show apparent aggravating effects on pancreatic injury or overall mortality in AP mice, it notably exacerbated pancreatic infection (Figure 6B and Supplemental Figure 10, A–C). These findings suggested that KA may play a role in leading to infections in AP. To explore the underlying mechanism, we evaluated the systemic and intestinal immune cells in these mice, which revealed a significant increase in the population of Tregs upon KA administration (Figure 6, C and D, and Supplemental Figure 10, D and E). Additionally, although KA did not directly affect the intestinal Th17 cell population, it did lower the intestinal Th17/Treg ratio (Supplemental Figure 10F). Tregs are recognized for their role in promoting immunosuppression in AP and hindering the effective antimicrobial response, thereby contributing to the development of ICs in AP (21). Using in vitro assays, we observed that KA not only facilitated the formation of Tregs in the Th17 differentiation system but also directly increased Treg levels in the Treg induction environment (Figure 5A and Figure 6E). Furthermore, the plasma and intestinal tissue levels of IL-10, an immunosuppressive cytokine, increased in AP mice following KA treatment (Supplemental Figure 10, G and H). KA treatment did not elevate plasma D-lac levels, suggesting its role operates independently of enhancing gut permeability (Supplemental Figure 10I). These findings indicated that KA may influence pancreatic infections by intensifying Treg-related immunosuppression.

Next, we evaluated the association between KA levels and immunosuppression among patients with AP. We assessed their plasma levels of the immunosuppression marker IL-10, inflammatory marker IL-6, and D-lac (22, 23). Among these indicators, IL-10 stood out as it was not only remarkably elevated in patients with ICs but also significantly correlated with both hospital stay duration and ICU stay length (Figure 6, F–I, and Supplemental Figure 11, A and B). These results highlighted the crucial role of immunosuppression in the development of ICs in AP. Importantly, among these 3 indicators, KA showed the strongest correlation with IL-10 levels, in both the discovery and validation patient groups (Figure 6J and Supplemental Figure 11, C–E). Based on these findings, we propose that KA may promote the development of ICs in AP via immunosuppression.

Finally, considering the detrimental effects of increased KA level and decreased Ser metabolites on the progression of AP, we hypothesized that interventions aimed at restoring tryptophan metabolism could have therapeutic effects. The metabolomic data revealed an inverse correlation between plasma levels of Kyn metabolites and Ser metabolites (Supplemental Figure 12A). The abundance of KA in the discovery group also exhibited inverse correlations with fecal Ser and Ind metabolites (Supplemental Figure 12B). These findings suggested that the competition for tryptophan resources, favoring Kyn production, contributes to the imbalanced tryptophan metabolism in AP. To address this imbalance, we utilized epacadostat (EP), an inhibitor of the indoleamine 2,3-dioxygenase 1 (IDO1) enzyme responsible for Kyn production (24). Treatment of AP mice with EP successfully restored intestinal Ser and 5-HTP levels while effectively inhibiting intestinal Kyn levels and plasma KA levels (Figure 6K and Supplemental Figure 12C). Furthermore, although EP treatment had minimal impact on the occurrence of pancreatic infections, it significantly reduced the pancreatic bacterial load (Figure 6, L and M) and significantly improved the 72-hour mortality (Figure 6N). In summary, addressing dysregulation in the tryptophan metabolism pathway holds promise as a potential therapeutic approach for the treatment of AP.

## Discussion

Using metabolomic tools, our recent study revealed notable changes in tryptophan metabolism in AP, characterized by decreased intestinal Ser and Ind metabolites, alongside an increase in Kyn metabolite levels. In fact, this alteration pattern in tryptophan metabolism has been previously reported in other acute or chronic inflammatory conditions, such as sepsis, as illustrated in our prior research (25), as well as in inflammatory bowel disease and COVID-19 (26–28). One plausible explanation is that increases in inflammatory cytokines such as IL-6 and IFN-γ, acting as IDO1 enzyme stimulators (29), accelerate the conversion of tryptophan to Kyn, which could then lead to reduced levels of Ser derivatives and depletion of tryptophan. Moreover, it is worth noting that the disturbed tryptophan metabolism was observed to be strongly associated with the susceptibility to ICs in AP. Our investigations revealed the elevated levels of plasma Kyn metabolites, particularly KA and 3-HK, were reliable predictors of infectious outcomes, particularly for multisite infections and IPN, in AP. While Kyn levels or the Kyn/Trp ratio have been linked to the occurrence of IPN previously (30), they were comparable between patients with and without ICs in our current study cohort (Supplemental Figure 4, E and F). Interestingly, we found that individuals with SAP exhibited increased Kyn/Trp or Kyn levels, but not 3-HK or KA levels (Supplemental Figure 3, E and F), suggesting that Kyn/Trp ratio and kynurenines may be more closely related to the degree of inflammation or organ failure, while 3-HK and KA may be more useful in indicating the risk of infection. Overall, our research highlights the diagnostic potential of tryptophan metabolic profiling in AP. The correlation of these metabolites with ICs could serve as a valuable tool for early identification of patients with AP at heightened risk of secondary infections, potentially improving the management of antibacterial therapies and surgical interventions in AP.

The strong clinical association observed also suggests a potential role of tryptophan metabolites in influencing bacterial translocation. Although the involvement of tryptophan metabolites in modulating T cell responses has been predominantly studied in the tumor microenvironment (24), our results highlight their roles in combating invading pathogens in the setting of a critical illness like AP. In this study, we demonstrated that Ser and 5-HTP, which enhance Th17 responses, have the potential to suppress infection. Conversely, KA, which promoted the generation of Tregs in this study, might potentially aggravate infection. Interestingly, some studies reported that KA alleviated AP by suppressing inflammatory neutrophils, while Ser has been found to play detrimental roles in certain acute inflammatory conditions (31, 32). Indeed, some researchers have suggested that maintaining a delicate balance in immune activation is crucial during critical illnesses, which is essential to prevent inflammatory damage while avoiding immunoparalysis when encountering pathogens (33, 34). We hypothesized that KA might play a dual role in AP, inhibiting inflammation but also impairing effective immune responses. This could explain the lack of obvious adverse effects of KA administration on pancreatic injury and mortality in our murine experiments. Understanding the specific receptors of these metabolites could help illuminate their functions in different pathogenic processes of AP. The aryl hydrocarbon receptor, which detects tryptophan metabolites and influences Th17 activity, may mediate the immunomodulatory effects of Ser and KA (35). Additionally, Ser receptors and the KA-sensing GPR35 receptor may also play roles in regulating T cell responses (36, 37). Further investigation into the complex mechanisms underlying these signaling pathways is imperative.

Other tryptophan metabolites demonstrating notable correlations with the progression of AP also merit our attention. For instance, we found that 3-HK was associated with a worsened outcome in AP, aligning with previous findings suggesting that Kyn monooxygenase enzymes and their product, 3-HK, exacerbate AP organ failure (38, 39). Several Ind derivatives, such as IAA and IPA, were decreased in the intestine and exhibited negative correlations with disease progression. This could be related to their known roles in enhancing the epithelial barrier and antimicrobial effects against pathogens (40, 41). Remarkably, we found increased levels of indole-3-lactic acid (ILA), a suppressor of Th17 cells (42), in plasma samples from both humans and mice with AP (Figure 2, B and C, and Supplemental Figure 3G). Further investigations are necessary to elucidate the source of ILA and its specific roles in the pathogenesis of AP. We proposed 2 potential explanations for the origin of ILA. ILA might be derived from the gut microbiota, easily translocating into the plasma under conditions of barrier disruption in AP. Alternatively, ILA could arise from host metabolism, with the IL4i1 enzyme converting tryptophan to KA and ILA (43), collectively potentially intensifying the immunosuppressive state in AP.

Our study also revealed alterations in the gut microbiota during AP. Consistent with previous research, we found that *Enterobacteriaceae* and *Enterococcaceae* were predominant taxa in AP (44). *Enterobacteriaceae* showed a particularly significant association with the occurrence of ICs in AP, consistent with the common involvement of pathogenic members of *Enterobacteriaceae*, such as *Escherichia-Shigella* and *Klebsiella*, in secondary infections in AP (4). Indeed, previous studies highlighted that *Escherichia-Shigella* worsened gut inflammation during AP (45), which may explain the association between *Enterobacteriaceae* and the development of infections. However, our correlation analysis of microbial taxa and metabolites showed minimal correlation between *Enterobacteriaceae* and these metabolites, indicating that *Enterobacteriaceae* may exacerbate AP directly, rather than indirectly through metabolites (Supplemental Figure 13, A and B). *Enterococcaceae*, another prevalent taxon in AP, has been linked to an elevated risk of secondary infection or mortality in ICU patients (46). Although we did not observe such associations in our cohort of patients with AP, we did find negative correlations of *Enterococcaceae* with various tryptophan metabolites (Supplemental Figure 13B). The potential influence of *Enterococcaceae* and its impact on gut metabolism in the progression of AP warrant further investigation.

This study has several limitations that should be considered. First, the study was conducted using a single set of samples; thus, the potential predictive value of Kyn metabolites for ICs should be validated in a larger sample size and through multicenter research. Second, it is important to note that the influence of Th17 cells in disease is multifaceted, encompassing both exacerbation of inflammatory injury and protection against invading pathogens, which is influenced by environmental signals and effector cytokines (19). Further investigations are necessary to elucidate the precise localization of the proposed protective Th17 cells and identify the effector cytokines released by these Th17 cells in AP. Third, it is important to acknowledge that our study primarily focused on the immunomodulatory roles of tryptophan metabolites on T cells, without investigating their potential impacts on other immune cells or nonimmune cells in AP. For instance, Ser has known effects on gut motility and the central nervous system that could also influence the progression of AP (47). Further investigations are indeed necessary to gain a comprehensive understanding of the mechanisms underlying the effects of these metabolites on AP.

In conclusion, our current study integrates untargeted and targeted metabolomic mass spectrometry analyses to explore the intricate interplay between gut metabolism and ICs in AP. We have identified altered tryptophan metabolism as a key feature in AP, closely linked to the development of ICs. Specifically, the diminished Ser pathways and heightened Kyn levels not only serve as potential indicators of future infections but also play a crucial role in fostering infections through immunomodulatory effects on Th17 cells/Tregs (Supplemental Figure 14). These findings highlight plasma biomarkers for predicting AP outcomes and provide valuable insights for developing strategies targeting metabolism to address secondary infections following AP.

## Methods

### Sex as a biological variable.

Given that the majority of patients in our clinical study were male (60 male vs. 32 female) and no significant sex-based differences were observed in AP presentation (Supplemental Table 5), we used male mice for our experiments. Furthermore, our research center has extensive experience with male mouse models, and prior studies have not reported significant differences in AP outcomes between sexes. Since no significant differences were observed in the main clinical characteristics between sexes in patients with AP (Supplemental Table 5), we infer that the findings observed in male mice are likely applicable to females as well.

### Study design.

This descriptive, observational study of adult patients diagnosed with AP was conducted in the ICU of Ruijin Hospital, Shanghai, China, from November 2019 to December 2021. Exclusion criteria included gastroenterological diseases, a history of immunodeficiency, ongoing immunosuppressive therapy, hospital stay of less than 1 week, and nonpancreatic infections. A total of 92 patients with AP who met the inclusion criteria were included. Age- and sex-matched HCs, without gastrointestinal diseases and recent antibiotic treatment, were randomly selected from routine physical examinations. Plasma and stool samples collected at admission were initially stored at –20°C and then transferred to –80°C within 12 hours for preservation. To explore potentially important metabolic pathways in AP, untargeted LC-MS/MS and fecal 16S rRNA-sequencing analyses of samples from a subgroup of patients with AP (*n* = 36) were undertaken. Due to the failure to obtain both fecal and plasma samples from some patients, we analyzed only 33 fecal samples and 34 plasma samples from the discovery group. A validation set of plasma samples from the remaining patients with AP (*n* = 56) underwent targeted tryptophan metabolomic analysis. One patient was discharged early and therefore was excluded from the study of the correlation with length of hospital stay. The clinical characteristics of the 2 AP subgroups were comparable (Supplemental Table 1).

Demographic data and clinical characteristics of the patients were meticulously recorded. Severity quantification on day 0 of admission utilized the following parameters: BISAP score, APACHE II score, serum PCT level, severity classification (moderate, moderately severe, severe), duration of hospital stay, and duration of ICU stay. All ICs were diagnosed by experienced clinicians. Among this cohort, the most common ICs observed were bacteremia, pneumonia, intra-abdominal infection, and urinary infection. Bacteremia was defined as a positive blood culture sample. Pneumonia was defined on the basis of chest radiography showing infiltrative abnormalities with positive sputum culture, and intra-abdominal infection was defined on the basis of infected ascites, indicated by a positive bacterial culture of abdominal fluid. Urinary infection was defined as a positive bacterial culture of midstream urine. IPN was defined as a positive culture of peripancreatic fluid along with the observation of pancreatic necrosis during initial percutaneous drainage or surgical intervention. Patients with infections in more than 2 sites were defined as having multiple infections.

### Mouse model of AP.

Male C57BL/6 mice, aged 8 weeks, were acclimated under standard conditions with a 12-hour light/12-hour dark cycle for at least 72 hours prior to modeling. The mice were anesthetized with an i.p. injection of ketamine (100 mg/kg body weight) and xylazine (5 mg/kg body weight) before undergoing surgical procedures. The necrotizing AP model was induced by retrograde injection of TCA into the pancreatic bile duct at a rate of 5 μL/min for 10 minutes per 20 g body weight as van den Berg et al. described (8). After 24 hours, the mice were sacrificed for bacteriological analysis of pancreatic tissue. For the exacerbation tests involving KA and GSK805, 2% sodium taurocholate was used, while for the alleviation tests with 5-HTP and EP, 3% sodium taurocholate was used. The HC mice were anesthetized and underwent abdominal surgery, during which the duodenum was exposed, then put back and sutured in place.

To investigate the effects of specific treatments, the following compounds were administered at 4 hours after AP induction: 200 mg/kg 5-HTP via oral gavage, 50 mg/kg KA via i.p. injection, and 50 mg/kg EP via i.p. injection. Additionally, the RORγt inhibitor GSK805 (10 mg/kg) was administered twice, once at 20 hours before AP induction and again at 4 hours after AP induction. For survival assessment, these treatments were administered via i.p. injection twice: once at 4 hours and again at 24 hours after AP induction. The vehicle group was treated with an equivalent dose of DMSO.

### Bacteriologic analysis of mouse tissue.

The pancreas and lungs of mice were aseptically excised, dissected, weighed, and promptly homogenized in sterile PBS. The homogenates were then cultured on Columbia blood agar plates at dilutions of 1:10 and 1:100 and incubated for 24 hours at 37°C. Colonies were enumerated for computation of CFU/mg of pancreatic tissue.

### Monocyte isolation from small intestinal lamina propria.

Intestinal lamina propria cells were isolated following a previously described protocol with some modifications (48). Briefly, tissues excised from the distal ileum were incubated with PBS supplemented with 1 mM dl-dithiothreitol and 30 mM EDTA for 20 minutes at 37°C. Subsequently, the tissues were cut into small pieces and digested in RPMI 1640 medium with 10% fetal bovine serum, 100 U/mL collagenase, and 150 μg/mL deoxyribonuclease for 1 hour. The resulting cells were strained through 40 μm filters and separated in the 40%–80% Percoll interface via density gradient centrifugation.

### Primary CD4^+^ T cell isolation and polarization.

A 96-well plate was coated with 3–5 μg/mL of anti-mouse CD3. Naive CD4^+^ T cells were isolated from the spleens of 8-week-old male C57BL/6J mice. Spleens were homogenized in PBS, and the resulting suspension was filtered through a 70 μm cell strainer. Subsequently, primary spleen CD4^+^ T cells were isolated using the MojoSort Mouse CD4 T Cell Isolation Kit following the manufacturer’s guidelines. Cells were then plated at a density of 5 × 10^5^ cells per well in RPMI 1640 medium supplemented with 10% fetal bovine serum and 1% penicillin/streptomycin in 96-well plates.

For Th17 polarization, naive T cells were incubated with 5 μg/mL anti-mouse CD28, 100 ng/mL recombinant mouse IL-6, 1 ng/mL recombinant human TGF-β1, 5 ng/mL recombinant mouse IL-23, 5 ng/mL recombinant mouse IL-1β, 10 μg/mL anti-mouse IL-4, and 10 μg/mL anti-mouse IFN-γ for 4 days. Following polarization, Th17 cells were stimulated with cell activation cocktail (with Brefeldin A) for 4–5 hours and then stained for flow cytometry. For Treg induction, naive T cells were incubated with 3 μg/mL anti-mouse CD28, 5 ng/mL recombinant mouse IL-2, and 5 ng/mL recombinant human TGF-β1 for 5 days. After polarization, Tregs were directly stained for flow cytometry. The impact of metabolites was investigated by individually adding each metabolite to the medium at a concentration of 100 μM.

### Flow cytometry.

For flow cytometry analysis, 10^6^ cells were suspended in flow cytometry buffer containing PBS with 0.5% bovine serum albumin and 2 mM EDTA. After staining with the viability dye Zombie, surface markers were stained using the following antibodies: fluorescein isothiocyanate-labeled CD45 and peridinin-chlorophyll-protein-cyanine 5.5–labeled CD4. For transcription factor analysis, cells were fixed and permeabilized using a transcription factor staining buffer set for 30 minutes after surface marker staining, followed by incubation with the following antibodies: Alexa Fluor 647–labeled Foxp3, BV421-labeled T-bet, and PE-labeled RORγt. To detect cytokines, cells were stimulated with cell activation cocktail in RPMI 1640 medium for 5 hours, after which surface markers were labeled. Subsequently, cells were treated with fixation buffer and intracellular staining Perm/Wash Buffer solution as per the manufacturer’s protocols and stained with PE–IL-17A and BV421–IFN-γ antibodies. Splenocytes were acquired by passage through a 70 μm cell strainer, washed, and subjected to erythrocyte lysis using Lysis Buffer. Flow cytometric data were acquired using a FACSCanto II flow cytometer (BD Biosciences) and analyzed with Flowjo software version 10.0. The gating strategies and key resources can be found in Supplemental Methods.

### Statistics.

Statistical analyses were performed using SPSS version 16.0, GraphPad Prism version 10, and R software version 4.0.5. All bioinformatic analyses were carried out on the Majorbio Cloud Platform. Differences between 2 groups were appraised using Student’s 2-tailed *t* test or the Mann-Whitney *U* test, contingent on data normality. One-way ANOVA followed by Tukey’s correction was applied to assess differences between multiple groups. The χ^2^ test or Fisher’s exact test was used to compare proportions between 2 groups. Spearman’s or Pearson’s correlation analysis was performed based on data distribution normality. The diagnostic performance of biomarkers was assessed using ROC curves in GraphPad Prism. The significance threshold was set at *P* < 0.05.

### Study approval.

The study and protocols were approved by the Ethics Committee of Ruijin Hospital (no. ChiCTR1900022022). Written informed consent was secured from each participant. Animal procedures were conducted in compliance with the Animal Use and Care Committee of the Shanghai Committee on Animal Care. All surgical procedures were approved by the Institutional Animal Care and Use Committee at Shanghai Jiao Tong University.

### Data availability.

Detailed patient information is available in Supplemental Table 6. Metabolomic data are provided in Supplemental Tables 7 and 8. RNA-sequencing data have been deposited in the BioProject repository under accession number PRJNA1079084. Data presented in all graphs are included in the Supporting Data Values file. Detailed methods are shown in the Supplemental Methods.

## Author contributions

DW, SS, QZ, EM, YP, and DD designed and executed the experiments, interpreted data, and prepared the manuscript. DD, QZ, LM, DX, and WC assisted with statistical analysis and manuscript preparation. TS, LX, MG, YS, YZ, and Y Liu assisted with the animal and cell experiments. BZ, CJ, QN, YC, Y Lu, and QL contributed to the collection of clinical samples, related experimental data, and case records. The first 4 authors contributed equally to this work. DW performed the murine experiments and took primary responsibility for preparing the manuscript. SS collected clinical patient data and conducted multiomic analyses with assistance from BZ. QZ performed the in vitro experiments. The order of co–first authorship was determined based on the complexity of the tasks performed. All authors have reviewed and approved the final manuscript.

## Supplementary Material

Supplemental data

Supplemental table 6

Supplemental table 7

Supplemental table 8

Supporting data values

## Version 1 03/10/2025

Electronic publication
