# Supplementary material for: Metabolic shifts in tryptophan pathways during acute pancreatitis infections
Source: JCI Insight. 2025 Mar 10;10(5):e186745. doi: 10.1172/jci.insight.186745 (PMC11949050; doi:10.1172/jci.insight.186745)
Supplement: Supplemental data [file jciinsight-10-186745-s174.pdf]

Supplementary methods

Key resources table

REAGENT of RESOURCE	SOURCE	IDENTIFIER
**Antibodies**		
Anti-mouse CD3, clone 145-2C11	Biolegend	100340
Anti-mouse CD28, clone 37.51	Biolegend	102116
Anti-mouse IL-4, clone 11B11	Biolegend	504122
Anti-mouse IFN-γ, clone XMG1.2	Biolegend	505834
FITC anti-mouse CD45, clone 104	Biolegend	109806
PerCP/Cyanine5.5 anti-mouse CD4	Biolegend	100539
AF647 anti-mouse Foxp3, clone MF-14	Biolegend	126408
BV421 anti-mouse Tbet, clone 4B10	Biolegend	644832
PE anti-mouse RORγt, Q31-378	BD Pharmingen	562607
PE anti-mouse IL-17A, TC11-18H10.1	Biolegend	506903
BV421 anti-mouse IFNγ, XMG1.2	Biolegend	505830
Rabbit anti-mouse Zo-1	Service bio	GB111981
Rabbit anti-mouse Occludin	Service bio	GB111401
AF488-labeled goat anti-rabbit antibody	Service bio	GB25303
Cy3-labeled goat anti-rabbit antibody	Service bio	GB21303
**Chemicals, peptides, and recombinant proteins**		
Sodium taurocholate, TCA	Sigma-Aldrich	86339
Kynurenic acid, KA	Selleck	S4719
Serotonin HCl	Selleck	S4244
L-Kynurenine Hydrate	Aladdin	2922-83-0
5-Hydrotryptophan, 5-HTP	Sigma-Aldrich	H9772
Epacadostat, EP	Selleck	S9710
GS805	Selleck	S6767
RPMI 1640 medium	Gibco	72400047
Fetal Bovine Serum	Gibco	10099141
Penicillin/streptomycin	Gibco	15140122
DL-dithiothreitol, DTT	Sigma Aldrich	D9779
0.5 M Ethylenediaminetetraacetic acid, EDTA	BBI Life Science	B540625
Percoll	Sigma Aldrich	P1644
Bovine serum albumin, BSA	Beyotime	ST023
Collagenase	Sigma Aldrich	C9891
Deoxyribonuclease	Sigma Aldrich	DN25
Recombinant human TGF-β1	UA BIOSCIENCE	UA040085

Recombinant mouse IL-6	Biolegend	575704
Recombinant mouse IL-23	Biolegend	589002
Recombinant mouse IL-1β	Biolegend	575102
Cell activation cocktail, with Brefeldin A	Biolegend	423303
Recombinant mouse IL-2	Biolegend	575402
Fixation Buffer	Biolegend	420801
Intracellular Staining Perm Wash Buffer (10X)	Biolegend	421002
Zombie NIR Fixable Viability Kit	Biolegend	423106
Erythrocyte lysis buffer	Beyotime	C3702
Cell Lysis Buffer for Western and IP	Beyotime	P0013
RNAiso Plus	Takara	9109
TB Green Premix Ex Taq	Takara	RR420A
Columbia Blood Agar	Autobio, Zhengzhou	N/A
**Critical commercial assays**		
MojoSort Mouse CD4 T Cell Isolation Kit	Biolegend	480006
True-Nuclear Transcription Factor Buffer Set	Biolegend	424401
α-Amylase Assay Kit	Jiancheng Bioengineering Institute, Nanjing	C016
Lipase assay kit	Jiancheng Bioengineering Institute, Nanjing	A054
Human IL-10 Precoated ELISA Kit	Dayou	1111002
Human IL-6 Precoated ELISA Kit	Dayou	1110602
Mouse IL-10 Precoated ELISA Kit	Dayou	1211002
Enhanced BCA Protein Assay Kit	Beyotime	P0010
Colorimetric D-Lactate Assay Kit	AAT Bioquest	13811
Kynurenic Acid ELISA Kit	Abbexa	abx156754
PrimeScript RT Reagent Kit	Takara	RR036A
**Experimental Models: Organisms/Strains**		
C57BL/6 wild-type mice	Charles River	N/A
**Oligonucleotides**		
Mouse *Ifng*<br>Forward: ATGAACGCTACACACTGCATC<br>Reverse: CCATCCTTTTGCCAGTTCCTC	N/A	N/A
Mouse *Il17a*<br>Forward: TTTAACTCCCTTGGCGCAAAA<br>Reverse: CTTTCCCTCCGCATTGACAC	N/A	N/A
Mouse *Ocln*	N/A	N/A

Forward: TTGAAAGTCCACCTCCTTACAGA		
Reverse: CCGGATAAAAAGAGTACGCTGG		
Mouse *Cldn1*	N/A	N/A
Forward: GGGGACAACATCGTGACCG		
Reverse: AGGAGTCGAAGACTTTGCACT		
Mouse *Muc2*	N/A	N/A
Forward: TGTGGAACCGGGAAGATG		
Reverse: GACCACAGGTATGGTTCTGGA		
Mouse *Tjp1*	N/A	N/A
Forward: CTTCTCTTGCTGGCCCTAAAC		
Reverse: TGGCTTCACTTGAGGTTTCTG		
Mouse *Reg3g*	N/A	N/A
Forward: TTCCTGTCCTCCATGATCAAAA		
Reverse: CATCCACCTCTGTTGGGTTCA		
Mouse *Defa5*	N/A	N/A
Forward: CTAATACTGAGGAGCAGCCAGG		
Reverse: GCAGCCTCTTATTCTACAATAGCA		
Mouse *β-actin*	N/A	N/A
Forward: GGCTGTATTCCCCTCCATCG		
Reverse: CCAGTTGGTAACAATGCCATGT		
**Deposited data**		
RNA sequencing datasets	This paper	BioProject PRJNA1079084
**Software and algorithms**		
SPSS 16.0	N/A	N/A
GraphPad Prism 10	N/A	N/A
Majorbio Cloud Platform	N/A	N/A
Flowjo 10.0	N/A	N/A

Method details

Determination of amylase and lipase levels

Amylase and lipase levels in plasma were measured using α-Amylase Assay Kits and Lipase Assay Kits.

Determination of IL-6 and IL-10 levels

IL-6 and IL-10 levels in human plasma were determined using the Human IL-10 Precoated ELISA and Human IL-6 Precoated ELISA Kits, and IL-10 levels in mouse plasma using the mouse IL-10 Precoated ELISA Kit, in accordance with the manufacturer's instructions. Mouse ileal segments

were isolated, homogenized in 1 mL Cell Lysis Buffer for western blotting and immunoprecipitation, and then centrifuged at 12,000 × g for 5 min. The supernatants were collected and stored at -80°C. Protein concentrations were measured using the Enhanced BCA Protein Assay Kit. Ileal IL-10 levels were determined using the supernatants and standardized by protein concentration.

Determination of D-lac and KA levels

Colorimetric D-Lactate Assay Kit was used to determine D-lac concentrations. For the EP intervention experiment, the KA level was determined using the KYNA ELISA Kit, in accordance with the manufacturer's instructions.

Histological analysis

Mouse pancreatic tissue samples were gently rinsed with PBS to remove debris, followed by fixation in 4% paraformaldehyde. After a 24-h fixation period, the pancreatic tissues were processed for routine histology, sectioned, and stained with hematoxylin and eosin. Histopathologic scoring of pancreatic samples was conducted as previously described.(Van Laethem et al., 1995) Briefly, edema was graded on a scale of 0 to 3 (0: absent; 1: focal increase between lobules; 2: diffuse increase between lobules; 3: disruption and separation of acinar structures). Inflammatory cell infiltration was graded from 0 to 3 (0: absent; 1: present in ducts, around ductal margins; 2: present in the parenchyma, affecting < 50% of the lobules; 3: present in the parenchyma, affecting > 50% of the lobules). Acinar necrosis was graded on a scale of 0 to 3: 0: absent; 1: periductal necrosis, affecting < 5%; 2: focal necrosis, affecting 5%–20%; 3: diffuse parenchymal necrosis, affecting 20%–50%.

Immunofluorescence l analysis of Zo-1 and Occludin Expression

For the detection of Zo-1 and Occludin, tissue sections were deparaffinized, rehydrated, and subjected to antigen retrieval using EDTA buffer in a microwave. Sections were then outlined with a hydrophobic barrier to prevent antibody runoff, blocked with BSA solution, and incubated overnight at 4°C with anti-mouse Zo-1 or anti-mouse Occludin antibodies. Following washing, sections were incubated with Cy3-labeled goat anti-rabbit secondary antibody for Zo-1 and AF488-labeled goat anti-rabbit secondary antibody for Occludin at room temperature. Autofluorescence quenching, nuclear counterstaining with DAPI, and slide mounting with an anti-fade medium were subsequently performed. Fluorescence imaging was conducted using appropriate filter sets.

The staining resulted in blue nuclei with DAPI, green Occludin with FITC, and red Zo-1 protein with Cy3. The positive area ratio was quantified by calculating the red or blue area relative to the total tissue area.

RNA extraction and real-time quantitative PCR (qPCR)

Total RNA from tissue or cell samples was isolated using RNAiso Plus, and cDNA was synthesized using the PrimeScript RT Reagent Kit. Real-time qPCR was performed using TB Green™ Premix

Ex Taq™ on a LightCycler 480 Real-Time PCR system (Roche, Switzerland). β-actin served as the internal control for gene expression.

Flow cytometry gating strategies.

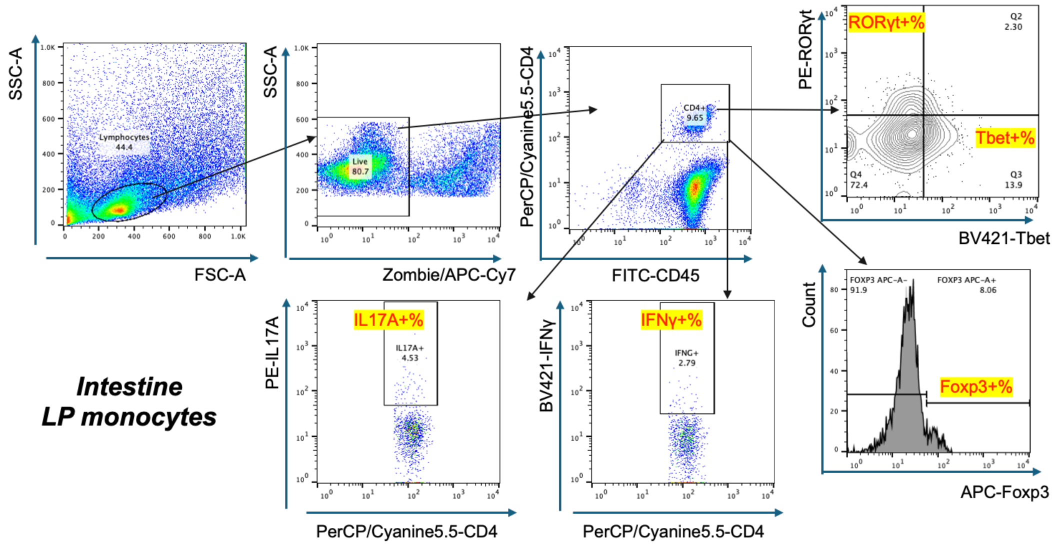

RNA sequencing

RNA derived from small intestine tissue was extracted as described above. An RNA sequencing transcriptome library was prepared using the TruSeq™ RNA sample preparation kit from Illumina (USA). Briefly, mRNA was isolated in accordance with the polyA selection method using oligo(dT) beads and then fragmented in fragmentation buffer. Double-stranded cDNA was synthesized using the SuperScript Double-Stranded cDNA Synthesis Kit (Invitrogen), employing random hexamer primers. Libraries were then size selected for target cDNA fragments of 300 base pairs by 2% Low Range Ultra Agarose electrophoresis, followed by PCR amplification using Phusion DNA polymerase (New England Biolabs, USA) for 15 PCR cycles. After quantification by TBS380, paired-end RNA sequencing was performed with the Illumina HiSeq xten/NovaSeq 6000 sequencer. The raw paired-end reads were trimmed, and the poor-quality reads were removed, by SeqPrep (https://github.com/jstjohn/SeqPrep) and Sickle (https://github.com/najoshi/sickle), respectively, with default parameters. Clean reads were separately aligned to the reference genome with the orientation mode using HISAT2 (http://ccb.jhu.edu/software/hisat2/index.shtml) software and assembled by StringTie (https://ccb.jhu.edu/software/stringtie/index.shtml?t=example). Gene abundance was quantified by RSEM (http://deweylab.biostat.wisc.edu/rsem/), and a differential expression analysis was performed using DEGs with |log2Fold Change (FC)|> 1. Differential gene expression analysis was performed using both the edgeR and DESeq2 R packages, and Kal's test with false discovery rate correction was applied. After Benjamini-Hochberg post hoc correction, an adjusted P value of < 0.05 was used to identify DEGs. Functional groups of DEGs were analyzed for involvement in Kyoto Encyclopedia of Genes and Genomes (KEGG) pathways. All bioinformatic analyses were performed using the Majorbio Cloud Platform.

Untargeted metabolome analysis through LC-MS/MS

Metabolite extraction

Each 50-mg fecal sample was accurately weighed for extraction of metabolites using a 400 μL methanol solution in water (4:1, v/v), with 0.02 mg/mL L-2-chlorophenylalanin as internal standard. The mixture was allowed to settle at -10°C, homogenized with a high-throughput tissue crusher (Wonbio-96c; Shanghai Wanbo Biotechnology Co., Ltd.) at 50 Hz for 6 min, then subjected to ultrasonication at 40 kHz for 30 min at 5°C. The samples were placed at -20°C for 30 min to precipitate proteins. After centrifugation at 13000 × g at 4°C for 15 min, each supernatant was carefully transferred to a sample vial for LC-MS/MS analysis.

Each 100-μL plasma sample was extracted using a 400-μL methanol:acetonitrile (1:1, v/v) solution. The mixture was sonicated at 40 kHz for 30 min at 5°C, then placed at -20°C for 30 min to precipitate proteins. After centrifugation at 13000 × g at 4°C for 15 min, the supernatant was carefully transferred to a new microtube and evaporated to dryness under a gentle stream of nitrogen. For ultra-high-performance liquid chromatography (UHPLC)-MS/MS analysis, the samples were reconstituted in a 100 μL loading solution of acetonitrile:water (1:1, v/v) via brief sonication in a 5°C water bath. Extracted metabolites were spun for 15 min at 13000 × g at 4°C on a bench-top centrifuge, and cleared supernatants were transferred to sample vials for LC-MS/MS analysis.

As a part of the system conditioning and quality control process, a pooled quality control (QC) sample representative of the entire sample set was prepared by mixing equal volumes of all samples. The QC samples were tested in the same manner as the analytic samples and were injected at regular intervals (every 10 samples) to monitor the stability of the analysis.

UHPLC-MS/MS analysis

The instrument platform for this analysis was the UHPLC-Q Exactive HF-X system (Thermo Fisher Scientific). Chromatographic conditions were as follows. A 2-μL sample was separated on an HSS T3 column (100 mm length × 2.1 mm inner diameter; 1.8 μm particle size) for MS detection. The mobile phases consisted of 0.1% formic acid in water:acetonitrile (95:5, v/v; solvent A) and 0.1% formic acid in acetonitrile:isopropanol:water (47.5:47.5:5, v/v; solvent B). The solvent gradient conditions were as follows: from 0 to 3.5 min, 0% B to 24.5% B (0.4 mL/min); from 3.5 to 5 min, 24.5% B to 65% B (0.4 mL/min); from 5 to 5.5 min, 65% B to 100% B (0.4 mL/min); from 5.5 to 7.4 min, 100% B to 100% B (0.4 mL/min to 0.6 mL/min); from 7.4 to 7.6 min, 100% B to 51.5% B (0.6 mL/min); from 7.6 to 7.8 min, 51.5% B to 0% B (0.6 mL/min to 0.5 mL/min); from 7.8 to 9 min, 0% B to 0% B (0.5 mL/min to 0.4 mL/min); and from 9 to 10 min, 0% B to 0% B (0.4 mL/min) to equilibrate the systems. The sample injection volume was 2 μL and the flow rate was set to 0.4 mL/min. The column temperature was maintained at 40°C. During the period of analysis, all samples were stored at 4°C.

MS data were collected using a Thermo UHPLC-Q Exactive HF-X Mass Spectrometer equipped

with an electrospray ionization (ESI) source operating in either positive or negative ion mode. The following optimal conditions were set: heater temperature, 425°C; capillary temperature, 325°C; sheath gas flow rate, 50 arb; aux gas flow rate, 13 arb; ion-spray voltage floating (ISVF), -3500 V in negative mode and 3500 V in positive mode; normalized collision energy, 20-40-60 V rolling for MS/MS. Full MS resolution was set to 60000, and MS/MS resolution was set to 7500. Data acquisition was performed with the Data Dependent Acquisition (DDA) mode. Detection was carried out over a mass range of 70–1050 m/z.

Data preprocessing and annotation

After the MS detection was completed, the raw LC/MS data were preprocessed by Progenesis QI (Waters Corporation, USA) software, and a three-dimensional data matrix was exported in CSV format. The information in this three-dimensional matrix included sample information, metabolite name, and mass spectral response intensity. Internal standard peaks, as well as any known false-positive peaks (including noise, column bleed, and derivatized reagent peaks), were removed from the data matrix, subjected to deredundancy, and peak pooled. Searching and identification of the metabolites were primarily conducted using the HMDB (http://www.hmdb.ca/), METLIN (https://metlin.scripps.edu/), and Majorbio databases.

Database search data were uploaded to the Majorbio cloud platform (https://cloud.majorbio.com) for data analysis. Metabolic features detected in ≥ 80% of any set of samples were retained. After filtering, minimum metabolite values were imputed for specific samples in which the metabolite levels fell below the lower limit of quantitation, and each metabolic feature was normalized by sum. To reduce the errors caused by sample preparation and instrument instability, the response intensity of the sample mass spectrum peaks was normalized by the sum normalization method, producing a normalized data matrix. At the same time, variables with a relative standard deviation (RSD) > 30% in QC samples were removed, and log10 algorithmizing was performed to obtain the final data matrix for subsequent analysis.

Differential metabolite analysis

Variance analysis of the matrix file was performed after data preprocessing. The R package ropls (Version 1.6.2) performed principal component analysis and orthogonal least partial squares discriminant analysis (OPLS-DA), and used 7-cycle interactive validation to evaluate the stability of the model. Student's t-test and fold-difference analysis were also performed. The selection of significantly different metabolites was determined on the basis of the variable importance in projection (VIP) obtained from the OPLS-DA model and the P value from the Wilcox's t-test; metabolites with VIP > 1 and P < 0.05 were considered significantly differential.

Differential metabolites among two groups were summarized and their biochemical pathways were mapped through metabolic enrichment and pathway analysis at KEGG (http://www. genome.jp/kegg/). The metabolites were classified by pathway involvement or function. Enrichment

analysis was usually applied to a group of metabolites in function node, whether or not each appeared in that node. The principle for this approach is that the annotation analysis of a single metabolite has evolved into group annotation analysis. The scipy.stats (Python package; https://docs.scipy.org/doc/scipy/) was exploited to identify statistically significantly enriched pathways using Fisher's exact test.

All bioinformatic analyses were carried out on the Majorbio Cloud Platform.

Targeted tryptophan metabolome analysis

Preparation of standard solutions and metabolite extraction

The 33 standards in the tryptophan pathway were accurately weighed for the preparation of standard solutions using 50% methanol. Appropriate amounts of the above solutions were taken to prepare a mixed standard, which was diluted to prepare different concentrations.

A 25-mg fecal sample was weighed precisely in a tube, to which 10 µL internal standard solution (Trp-D5, 4000 ng/mL) and 190 µL extraction solution (methanol:water = 4:1) were added. The mixture was homogenized at -10°C using a high-throughput tissue crusher (Wonbio-96c) at a frequency of 50 Hz for 6 min, then sonicated at 40 kHz for 30 min at 5°C. The sample was placed at -20°C for 30 min and centrifuged at 13000 × g at 4°C for 15 min, following which the supernatant was injected into the LC-MS/MS system for analysis.

A 100-µL plasma sample, 10 µL internal standard solution (Trp-D5, 4000 ng/mL) and 990 µL extraction solution (acetonitrile:water = 9:1) were taken into a 1.5-mL centrifuge tube. The mixture was vortexed for 30 s, sonicated at 40 kHz for 30 min at 5°C, placed at -20°C for 30 min, and centrifuged at 13000 × g at 4°C for 15 min. The supernatant was injected into the LC-MS/MS system for analysis.

UHPLC-MS/MS analysis

Samples were analyzed on a Nexera Series LC-40 system coupled with a QTRAP® 6500+ mass spectrometer (Sciex, USA) at Majorbio Bio-Pharm Technology Co. Ltd. (Shanghai, China). Briefly, samples were separated on an ACQUITY UPLC® HSS T3 column (2.1 mm length × 150 mm inner diameter; 1.8 µm particle size) at 40°C. Separation of the metabolites was achieved at a 1 mL/min flow rate with a mobile phase gradient consisting of water containing 0.1% formic acid (solvent A) and 100% acetonitrile in water containing 0.1% formic acid (solvent B). The total chromatographic separation was 18 min. The elution gradient conditions were as follows: 0.0–2.5 min, 1–11% B; 2.5–5.5 min, maintain 11% B; 5.5–6.5 min, 11%–28% B; 6.5–7.5 min, maintain 28% B; 7.5–12.5 min, 28%–50% B; 12.5–13.5 min, 50%–95%B; 13.5–15.5 min, maintain 95%B; 15.5–15.6 min, 95%–1% B; 15.6–18 min, maintain 1% B. During the period of analysis, samples were stored at 4°C.

MS data were collected using a UHPLC coupled to a QTRAP® 6500+ mass spectrometer (Sciex,

USA) equipped with an ESI source operating in both positive and negative mode. The parameters were set as follows: source temperature, 550°C; curtain gas (CUR), 35 psi; CAD gas pressure, medium; both ion source Gas1 and Gas2, 50 psi; ISVF, 5500V/-4500V.

QC

QC samples comprising mixed samples or certain concentrations of mixed standard solutions were mainly used to assess the stability of the analytical system. QC samples were injected at regular intervals (every 10 samples), aiming for an RSD of these targets of < 15%.

Data processing

LC-MS raw data were imported into the Sciex software operating system. All ion fragments were automatically identified and integrated using default parameters. Manual verification was performed for all integrations. The metabolite concentration in each sample was calculated from a linear regression standard curve.

16S rRNA sequencing analysis of the microbiota

Genomic DNA from stool samples was extracted with the TIANamp Stool DNA kit (TIANGEN), following the manufacturer's protocol. Amplicons of the V4 region of the 16S rRNA gene were generated using the 338F/806R primer pair, and sequencing was carried out on an Illumina PE300 platform by Majorbio Bio-Pharm Technology Co. Ltd. Raw FASTQ files were demultiplexed using an in-house script, subjected to quality filtering with fastp version 0.19.6, and merged using FLASH version 1.211. The sequences obtained after demultiplexing were quality filtered using fastp (v0.19.6) and merged with FLASH (v1.2.11). The high-quality sequences were denoised using DADA2 in Qiime2 (version 2020.2) with recommended settings. The resulting denoised sequences, referred to as amplicon sequence variants (ASVs), were rarefied to 20,000 sequences per sample to minimize the impact of sequencing depth on diversity analysis. Taxonomic assignment of the ASVs was performed using the Naive bayes, Vsearch, or Blast consensus taxonomy classifier in Qiime2 and the SILVA 16S rRNA database (v138). Bioinformatic analysis of the gut microbiota was carried out using the Majorbio Cloud platform (https://cloud.majorbio.com). Linear Discriminant Analysis Effect Size was employed from the taxonomic phylum level to the genus level, incorporating varied thresholds for linear discriminant analysis scoring under distinct conditions. Correlations between the abundance of taxa and a clinical parameter were analyzed using Spearman correlation analysis, in which a P value < 0.05 was considered significant.

Table S1. Clinical and demographic characteristics of AP patients.

	AP N=92 (TOTAL)	AP N=36 (DISCOVERY)	AP N=56 (VALIDATION)	P
Age, years	43.8±13.7	45.7±12.7	42.5±14.3	0.274
Sex, male/female	60/32	25/11	35/21	0.495
Inducement				
Biliary	45 (48.9%)	19 (52.8%)	26 (46.4%)	0.552
Alcohol	16 (17.4%)	9 (25.0%)	7 (12.5%)	0.123
Hyperlipidemic	61 (66.3%)	25 (69.4%)	36 (64.3%)	0.609
IC[a] Occurrence	43 (46.7%)	19 (52.8%)	24 (42.9%)	0.352
IC Occurred at Multiple Sites[b]	19 (20.6%)	7 (19.4%)	12 (21.4%)	0.388
IPN[c]	12 (13.0%)	5 (13.9%)	7 (12.5%)	1.000
Severity				
Moderate AP	19 (20.7%)	6 (16.7%)	13 (23.2%)	0.450
Moderate Severe AP	24 (26.1%)	11 (30.6%)	13 (23.2%)	0.434
Severe AP	49 (53.3%)	19 (52.8%)	30 (53.6%)	1.000
Origin of Infection				
Pulmonary	35 (38.0%)	17 (47.2%)	18 (32.1%)	0.146
Urinary	14 (14.1%)	5 (13.9%)	9 (16.1%)	1.000
Abdominal	14(14.1%)	6 (16.7%)	8 (14.3%)	1.000
BISAP Score	2.59±0.81	2.50±0.91	2.64±0.74	0.414
APAHCE II Score	10.67±4.74	9.64±4.65	11.34±4.72	0.093
Serum PCT, ng/mL	5.38±7.34	5.44±7.35	5.35±7.40	0.955
Mortality within 90 days	3 (3.3%)	2 (5.6%)	1 (1.8%)	0.151
ICU stay, days	22.2±28.8	21.6±26.5	22.6±30.4	0.874
In hospital stay, days	35.1±28.7	36.5±24.7	34.2±31.1	0.709

The continual variables are presented as a format of average ± standard deviation, and the frequency variables are presented as a format of Number (Proportion). For

continuous variables, the comparison between the two groups was conducted using Student's t-test. For categorical variables, the comparison between the two groups was performed using either the Chi-square test or Fisher's exact test.

a. IC, Infectious Complications, b. IC occurred more than one site. c. IPN, infected necrosis pancreatitis.

Table S2 Comparison of clinical characteristics between AP patients with IC and AP patients without IC.

	AP-IC N=43	AP-non-IC N=49	P
Age	43.67±13.91	43.94±13.70	0.927
Gender(M/F)	28/15	32/17	0.985
BISAP Score	2.83±0.87	2.37±0.70	**0.005**
APACHE II Score	12.56±5.46	9.03±3.26	**< 0.001**
Serum PCT, pg/mL	8.14±9.03	2.96±4.24	**0.001**
Death	3(6.9%)	0 (0)	0.098
In hospital Stay, days	52.8±33.8	19.8±7.4	**< 0.001**
ICU stay, days	36.1±37.5	10.2±5.98	**< 0.001**
SAP (%)	32(74.4%)	17(34.7%)	**< 0.001**

The continual variables are presented as a format of average ± standard deviation, and the frequency variables are presented as a format of Number (Proportion).

For continuous variables, the comparison between the two groups was conducted using Student's t-test. For categorical variables, the comparison between the two groups was performed using either the Chi-square test or Fisher's exact test.

Table S3 Comparison of clinical characteristics between AP patients with IPN and AP patients without IPN.

	**AP-non-IPN N=80**	**AP-IPN N=12**	**P**
Age	42.5±12.1	44.0±14.0	0.724
Gender(M/F)	52/28	8/4	>0.999
BISAP Score	2.56±0.81	2.75±0.87	0.460
APACHE II Score	9.98±3.82	15.33±7.35	**0.029**
Serum PCT, pg/mL	4.43±6.45	11.72±9.84	**0.028**
Death	0 (0)	3(25%)	**0.002**
In hospital Stay, days	26.27±13.24	93.08±35.31	**<0.001**
ICU stay, days	12.84±7.94	83.92±39.26	**<0.001**
SAP (%)	37(46.25%)	12(100%)	**<0.001**

The continual variables are presented as a format of average ± standard deviation, and the frequency variables are presented as a format of Number (Proportion).

For continuous variables, the comparison between the two groups was conducted using Student's t-test. For categorical variables, the comparison between the two groups was performed using either the Chi-square test or Fisher's exact test.

Table S4 Comparison of clinical characteristics between AP patients with single-site IC and AP patients with multi-site IC.

	AP-IC (Single site) N=24	AP-IC (Multi sites) N=19	P
Age	44.1±14.2	43.1±13.9	0.815
Gender(M/F)	16/8	12/7	0.811
BISAP Score	2.79±0.88	2.89±0.88	0.705
APACHE II Score	10.21±3.09	15.53±6.37	**<0.001**
Serum PCT, pg/mL	9.37±9.04	7.18±9.09	0.436
Death	0	3(15.8%)	0.079
In hospital Stay, days	15.56±7.42	61.05±44.04	**<0.001**
ICU stay, days	34.9±13.3	74.5±38.4	**<0.001**
SAP (%)	14(58.3%)	18(94.7%)	**0.007**

The continual variables are presented as a format of average ± standard deviation, and the frequency variables are presented as a format of Number (Proportion).

For continuous variables, the comparison between the two groups was conducted using Student's t-test. For categorical variables, the comparison between the two groups was performed using either the Chi-square test or Fisher's exact test.

Table S5. Clinical and demographic characteristics of AP patients stratified by gender.

	AP-M N=60	AP-F N=32	P
Age	41.8±12.7	47.6±14.9	0.053
BISAP Score	2.57±0.79	2.63±0.87	0.745
APACHE II Score	10.53±5.50	9.69±4.11	0.448
Serum PCT, pg/mL	6.33±7.83	3.60±6.02	0.089
Death	2 (3.3%)	0 (0%)	0.541
In hospital Stay, days	34.2±29.5	36.8±27.3	0.684
ICU stay, days	21.8±29.7	23.0±27.5	0.842
IC (%)	28 (46.7%)	15 (46.9%)	0.985
IPN (%)	8 (13.3%)	4 (12.5%)	1.000
SAP (%)	35 (58.3%)	14 (43.8%)	0.182

The continual variables are presented as a format of average ± standard deviation, and the frequency variables are presented as a format of Number (Proportion).

For continuous variables, the comparison between the two groups was conducted using Student's t-test. For categorical variables, the comparison between the two groups was performed using either the Chi-square test or Fisher's exact test.

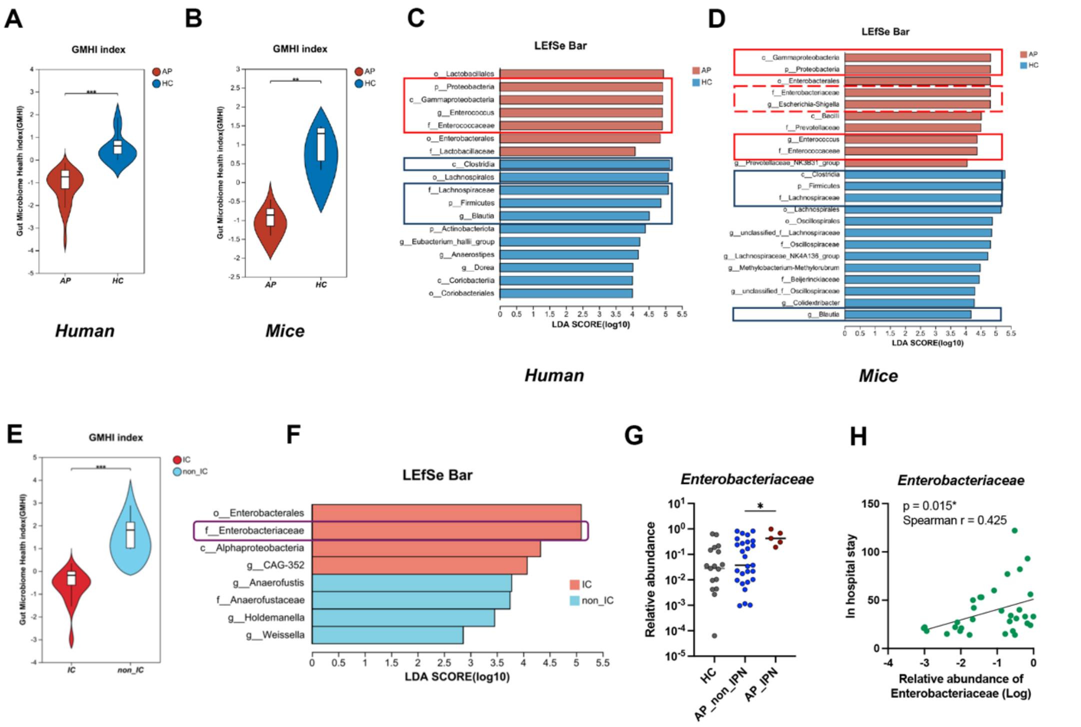

Figure S1. Alterations in the microbiota in AP.

(A-D) Microbiota alterations were observed in AP patients (n=32) and AP mice (n=12) compared to the healthy control (HC) group (n=19 for patients, n=5 for mice). Panels A and B represent the Gut Microbiota Health Index (GMHI). Panels C and D display the differential taxa identified using LefSe analysis from the phylum to genus level (p<0.05, LDA>4).

(E-F) Microbial features were compared between AP patients with the development of infected pancreatic necrosis (AP-IC, n=18) and AP patients without infected pancreatic necrosis (AP-non-IC, n=14). Panel E represents the GMHI index, while panel F shows the differential taxa identified using LefSe analysis from the phylum to genus level (p<0.05, LDA>2.5).

(G) The abundance of Enterobacteriaceae was compared among AP patients with infected pancreatic necrosis (n=5), AP patients without infected pancreatic necrosis (n=27), and the HC group (n=19). Analysis was performed using the Mann-Whitney test.

(H) Spearman correlation analysis was conducted to assess the correlation between Enterobacteriaceae abundance, and the length of hospital stay among AP patients (n=32). *P<0.05.

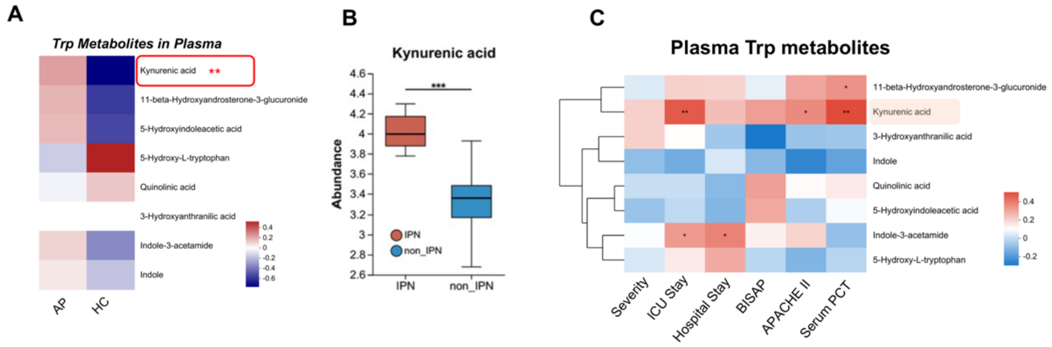

Figure S2 Untargeted plasma metabolome in the discovery group of AP.

(A) Heatmap illustrating the relative abundance of plasma tryptophan metabolites in the discovery group of AP patients and healthy controls (HCs).

(B) Relative abundance of plasma kynurenic acid (KA) levels in AP patients with infected pancreatic necrosis (IPN, n = 6) and those without (non-IPN, n = 28).

(C) Spearman correlation heatmap displaying the associations between clinical severity parameters and tryptophan metabolites annotated in plasma samples (n = 34) from AP patients. APACHE II, Acute Physiology and Chronic Health Evaluation II; BISAP, bedside index for severity in AP; PCT, procalcitonin.

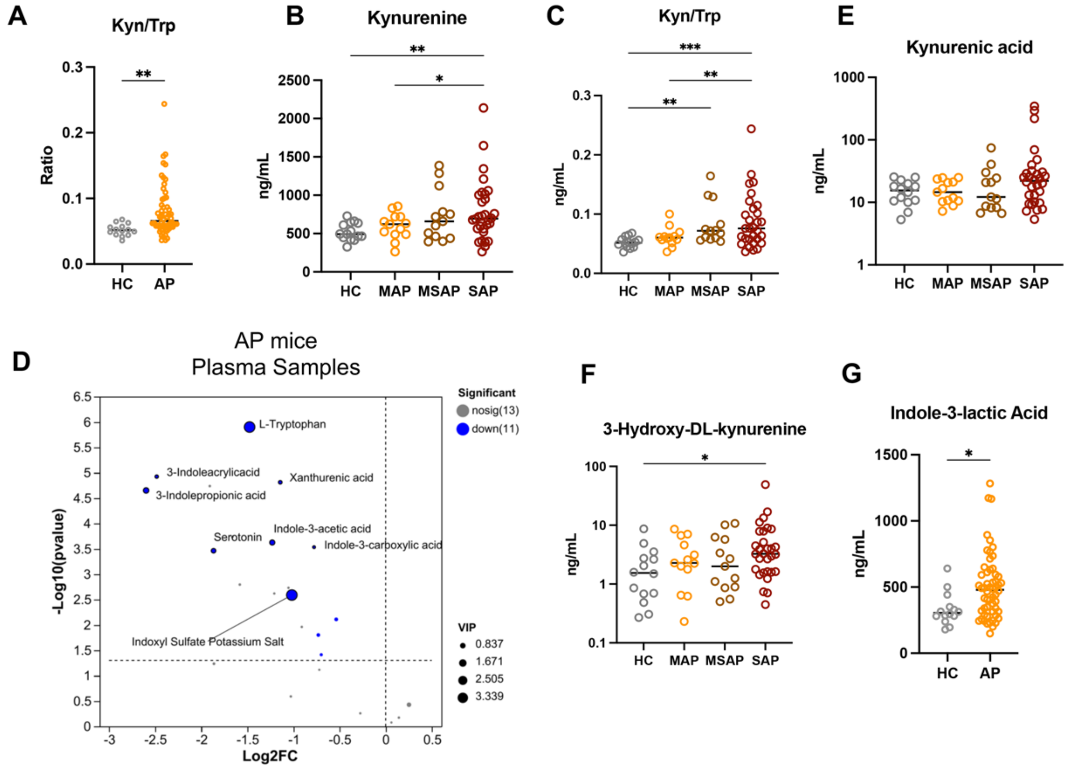

Figure S3 Plasma tryptophan metabolism in the validation group of AP

(A) Plasma kynurenine/tryptophan ratio (Kyn/Trp) levels in AP patients (n = 56) compared to healthy controls (HCs, n = 14), analyzed using Student's t-test.

(B, C, E, F) Comparison of kynurenine, kynurenine/tryptophan ratio, kynurenic acid, and 3-Hydroxy-DL-kynurenine levels among groups: healthy control (HC, n = 14), mild acute pancreatitis (MAP, n = 13), moderately severe acute pancreatitis (MSAP, n = 13), and severe acute pancreatitis (SAP, n = 30). Statistical analysis was conducted using Brown-Forsythe and Welch ANOVA tests.

(D) Volcano plot showing plasma metabolites with significantly different concentrations between AP-induced mice (n = 11) and HC mice (n = 5) using Student's t-test.

(G) Plasma levels of indole-3-lactic acid in AP patients (n = 56) versus HCs (n = 14), analyzed using Student's t-test.

*P < 0.05, **P < 0.01, ***P < 0.001.

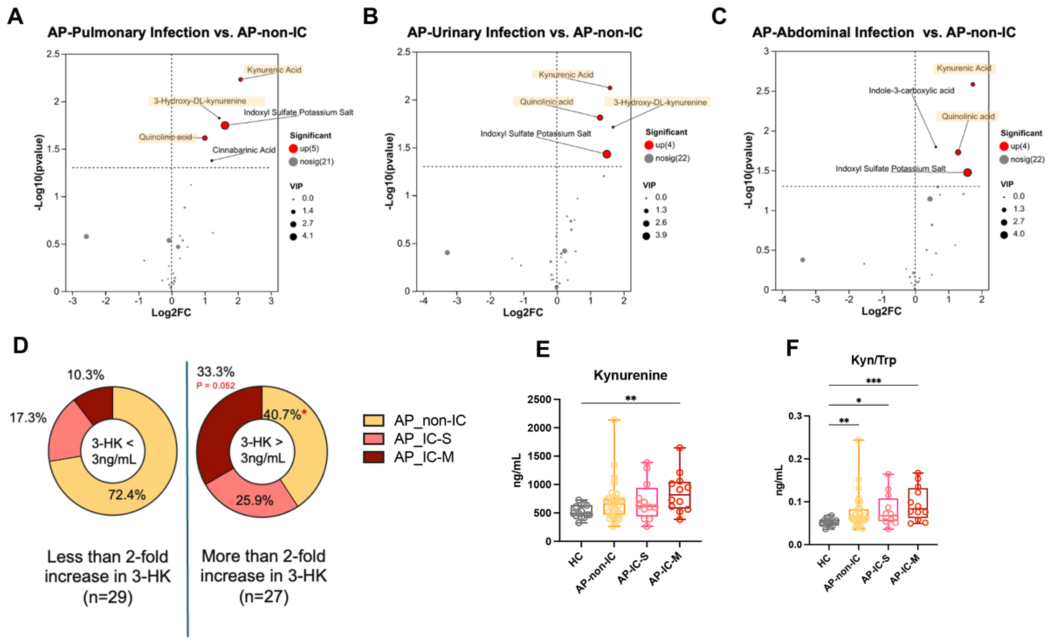

Figure S4 Plasma tryptophan metabolism in AP patients with infectious complications (IC).

(A-C) Volcano plot showing plasma metabolites with significantly different concentrations between AP-Pulmonary infection (n = 18), AP-Urinary infection (n = 9), AP-Abdominal infection (n = 8) and HC mice (n = 14) using Student's t-test.

(D) Distribution of multi-site IC, single-site IC, and non-IC among AP patients with 3-HK levels above or below 3 ng/mL (representing a 2-fold change relative to the healthy group). Ratios were compared using the Chi-square or Fisher's exact test.

(E-F) Plasma levels of the Kynurenine (E) and Kyn/Trp (F) in subgroups of AP patients without IC (AP-non-IC, n = 32), single-site IC (AP-IC-S, n = 12), or multi-site IC (AP-IC-M, n = 12), and HCs (n = 14). Data analyzed using the Kruskal-Wallis statistic.

*P < 0.05, **P < 0.01, ***P < 0.001.

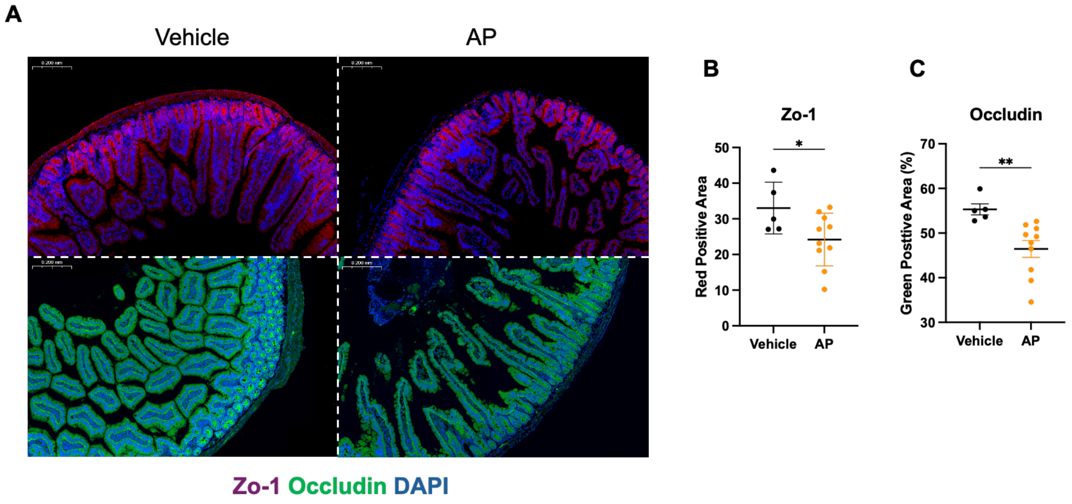

Figure S5. Intestinal barrier changes in AP.

(A) Representative immunofluorescent histological images of the duodenum showing the intestinal mucosa in AP. Zo-1 is stained in red, Occludin in green, and nuclei are stained blue with DAPI.

(B–C) Quantification of the positive area ratio of Zo-1 (B) and Occludin (C) in the duodenal tissues of Vehicle (n = 5) and AP (n = 10) mice. Data are presented as mean ± standard deviation. Statistical analysis was performed using Student's t-test. *$P < 0.05$. **$P < 0.01$.

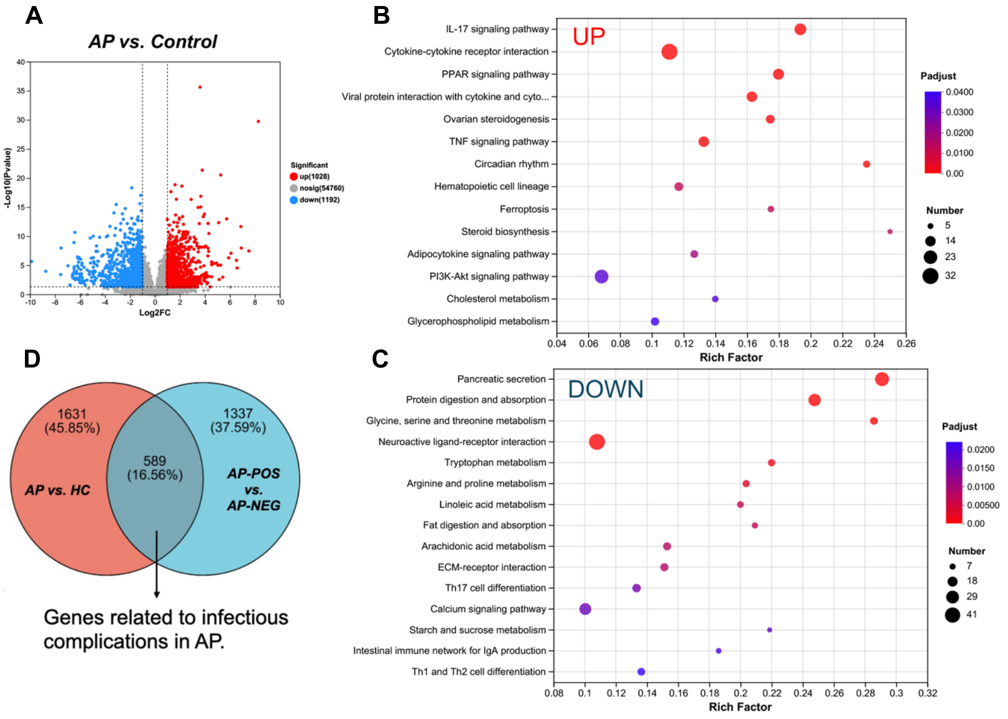

Figure S6 RNA-sequencing analysis of the small intestine in the AP.

(A) Volcano plot showing differentially expressed genes (DEGs) in the small intestine between AP (n = 10) and control (n = 5) mice. Blue and red dots represent depleted and enriched genes, respectively, with cut-offs of $P < 0.05$ and fold change > 2.

(B, C) Bubble diagrams illustrating Kyoto Encyclopedia of Genes and Genomes enrichment analysis of DEGs, with upregulated genes in AP shown in (B) and downregulated genes in AP shown in (C). Bubble size indicates the number of genes enriched in each pathway; color gradient reflects the significance of enrichment.

(D) Venn diagram of genes associated with infectious complications during AP identified through intersection of DEGs between the AP (n = 10) and healthy control (HC; n = 6) groups with those between the AP subgroups with (AP_POS; n = 5) and without (AP_NEG; n = 5) infectious complications.

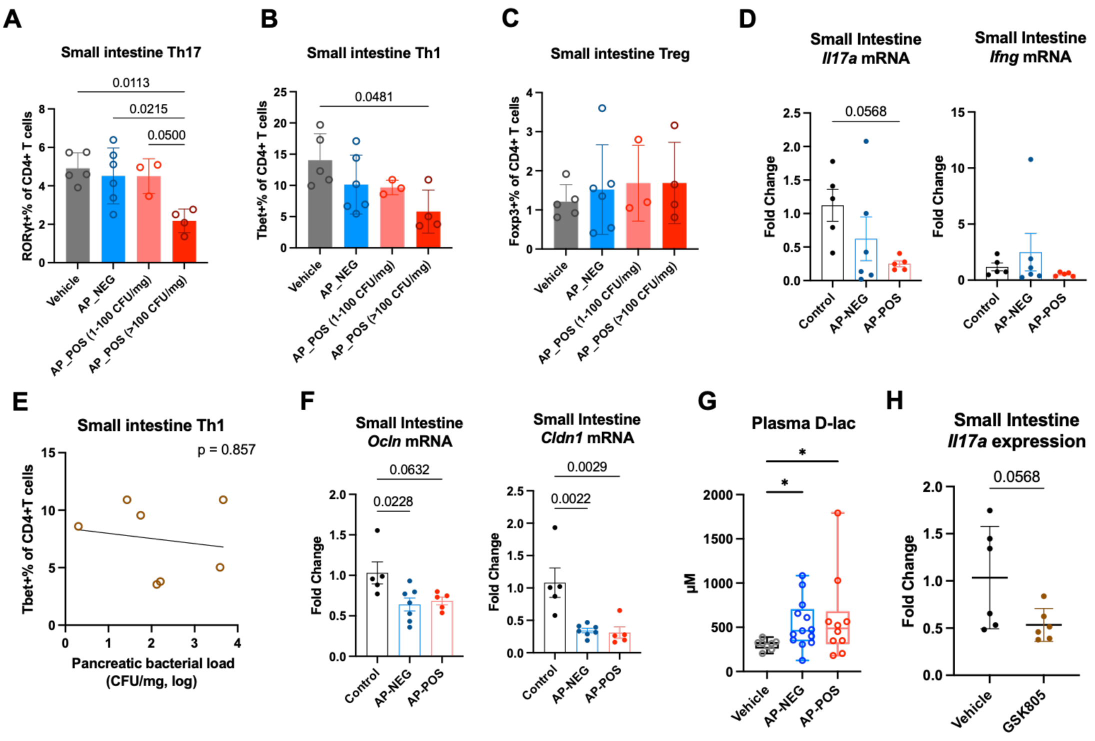

Figure S7. Immune and barrier changes in the small intestine of AP mice with pancreatic infection

(A–C) Flow cytometry analysis of Th17 cells (RORγt^{+}/CD4^{+}), Th1 cells (Tbet^{+}/CD4^{+}), and regulatory T cells (Tregs; Foxp3^{+}/CD4^{+}) in the small intestinal lamina propria of mice from the Vehicle (n = 5), AP-NEG (n = 6), AP-POS with low pancreatic bacterial load (1–100 CFU/mg, n = 3), and AP-POS with high pancreatic bacterial load (>100 CFU/mg, n = 4) groups. CFU, colony-forming units.

(D) mRNA expression levels of *Il17a* (F) and *Ifng* (G) in the small intestine of Vehicle (n = 5), AP-NEG (n = 6), and AP-POS (n = 5) group mice.

(E) Spearman correlation analysis showing the relationship between small intestinal Th1 cells and pancreatic bacterial load in AP mice (n = 7).

(F) mRNA expression levels of *Ocln* (F) and *Cldn1* (G) in the small intestine of Vehicle (n = 5), AP-NEG (n = 6), and AP-POS (n = 5) group mice.

(G) Plasma D-lactic acid (D-lac) levels in the Vehicle (n = 8), AP-NEG (n = 13), and AP-POS (n = 10) group mice.

(H) mRNA expression levels of *Il17a* in the small intestine of Vehicle and GSK805-treated AP mice (n = 6 per group).

Error bars represent the mean ± standard deviation (A–C, H) or mean ± standard error. Data were analyzed using one-way ANOVA with Tukey's test (A–C, F), the Brown-Forsythe ANOVA test with Dunnett's T3 multiple comparisons test (D), Kruskal-Wallis test (G), Student's t-test (H), or Spearman correlation test (E). *P < 0.05, **P < 0.01.

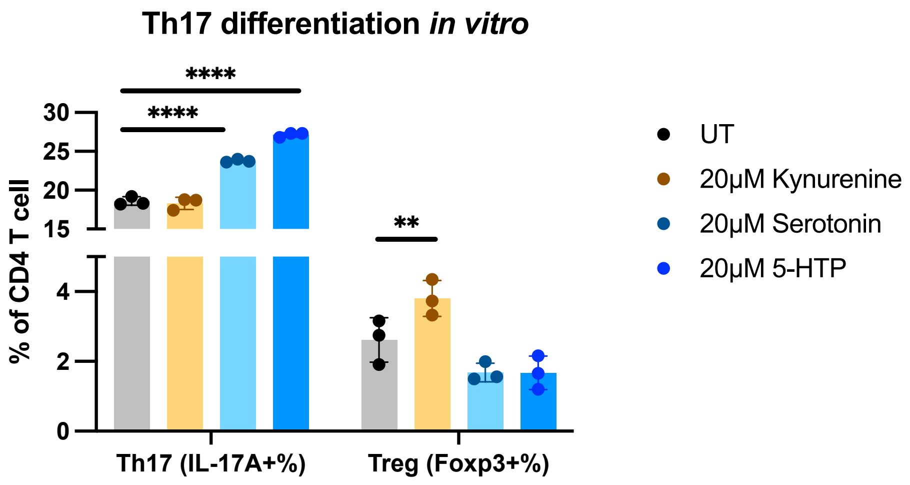

Figure S8. Impact of tryptophan metabolites on Th17 differentiation in vitro

Effects of 20 μM kynurenine, serotonin or 5-HTP on the polarization of murine primary spleen naïve CD4+ T cells towards Th17 compared with an untreated (UT) group (n = 3 per group). Treg, regulatory T cells.

Error bars represent the mean ± standard deviation, Data were analyzed using Student's t-test **P < 0.01, ****P < 0.0001.

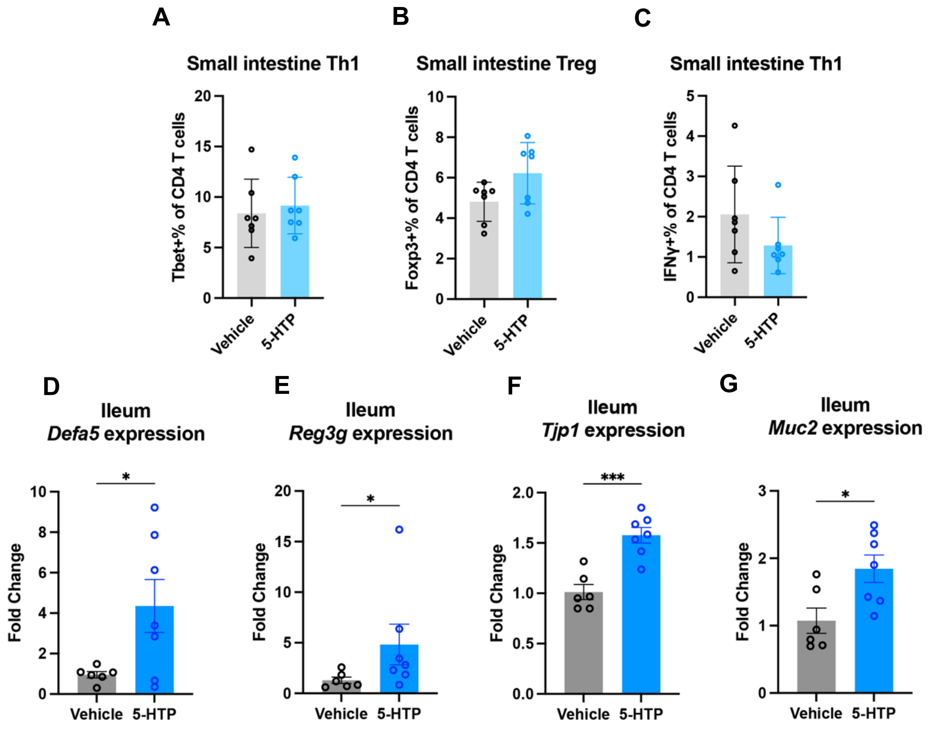

Figure S9 Effects of 5-hydroxytryptophan (5-HTP) supplementation on AP mice

(A–C) Flow cytometry analyses of the helper T (Th)1 cell and regulatory T cell (Treg) populations of small intestinal lamina propria in AP mice, with and without 5-HTP supplementation (n = 6–7 per group).

(D–G) mRNA expression levels of Defa5, Reg3g, Tjp1, and Muc2 in the small intestine of Vehicle- and 5-HTP-treated AP mice (n = 6–7 per group).

Error bars represent the mean ± standard deviation. Data analyzed using Student's t-test (A-C, D-F) or Mann-Whitney test (E); *P < 0.05, **P < 0.01.

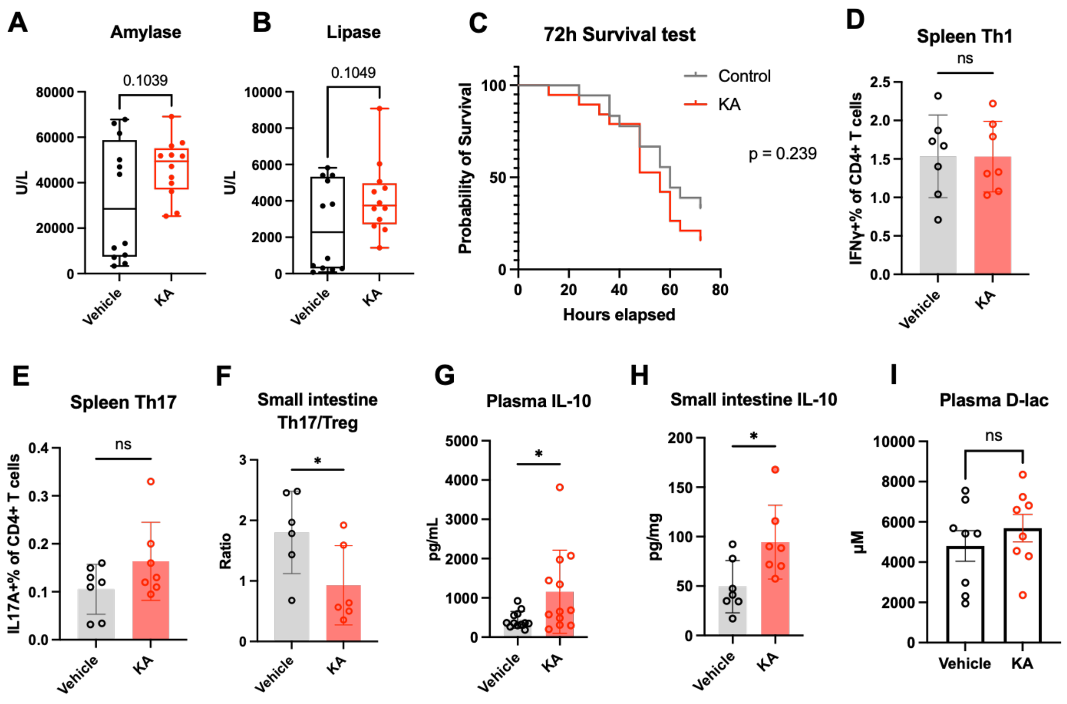

Figure S10 Influence of kynurenic acid (KA) administration on AP mice

(A, B) Levels of plasma amylase and lipase in AP mice administered KA or vehicle control (n = 12 per group).

(C) Survival curves of KA-treated AP mice (n = 22) and vehicle-treated mice (n = 23), analyzed by log-rank (Mantel-Cox) test.

(D–E) Proportions of helper T (Th) cells in the spleen of AP mice, with and without KA administration (n = 6–7 per group).

(F) Ratio of Th17 to regulatory T cells (Treg) in AP mice, with and without KA administration (n = 6 per group).

(G, H) IL-10 levels in plasma (G; n = 12) and ileum (H; n = 7) in the KA- and vehicle-treated groups.

(I) Levels of plasma D-lactic in AP mice administered KA or vehicle control (n = 8 per group). Data analyzed using Mann-Whitney test (A, B) or Student's t-test (D–I) *P < 0.05, **P<0.01, ns, not significant.

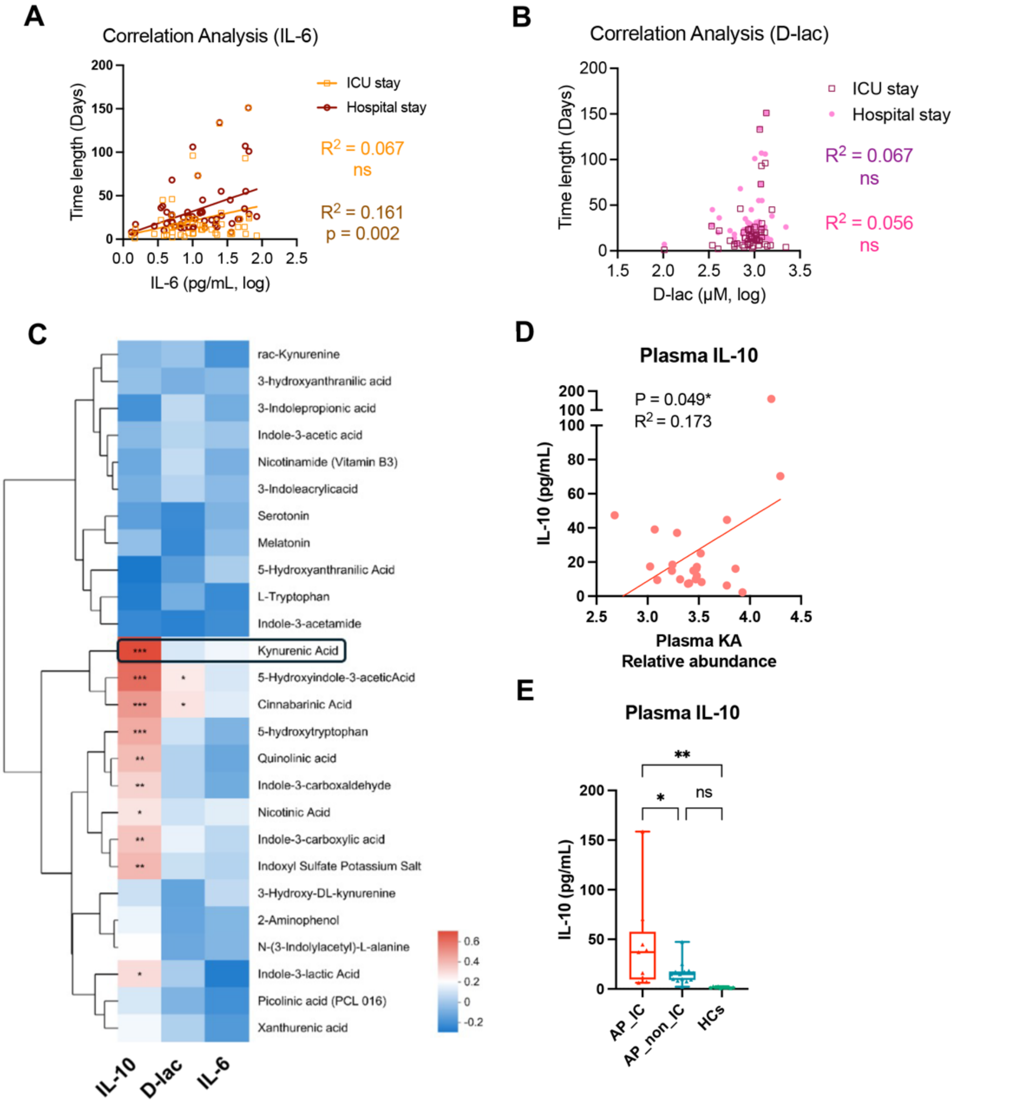

Figure S11. Associations between plasma indicators, kynurenic Acid (KA), and clinical outcomes in AP

(A–B) Pearson correlation analysis showing the relationships between plasma indicators (IL-6 in A and D-lac in B) and ICU stay duration and total hospital stay in AP patients (n = 55).

(C) Spearman correlation analysis between plasma indicators of immunosuppression and plasma levels of tryptophan metabolites among AP patients (n = 55). IL, interleukin; D-lac, D-lactic acid.

(D) Pearson correlation between the relative abundance of plasma KA and plasma IL-10 concentration among AP patients in the discovery group (n = 23).

(E) Plasma IL-10 levels among AP-infection patients (n=9), AP-non-infection patients (n=14), and HC (n=10) in the discovery group. Statistical analyses were conducted using one-way ANOVA with Tukey's test.

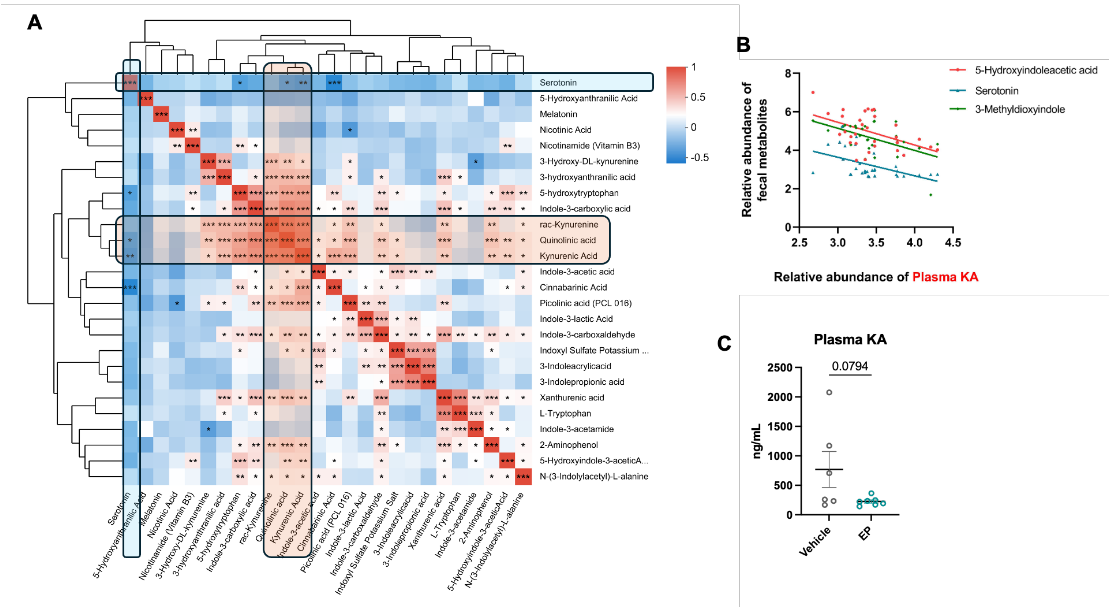

Figure S12 Correlation between the abundance of tryptophan metabolites in AP

(A) Spearman correlation analysis showing the abundance of each metabolite and the relationship between kynurenine metabolites and serotonin metabolites among AP patients (n = 55).

(B) Pearson correlation analysis of fecal levels of 5-hydroxyindoleacetic acid, serotonin, and 3-methyldioxyindole with plasma KA among AP patients (n = 31), all of which showed statistical significance.

(C) Abundance of plasma KA between AP mice with and without EP treatment (n = 6–7 per group). Data analyzed using Student's t-test.

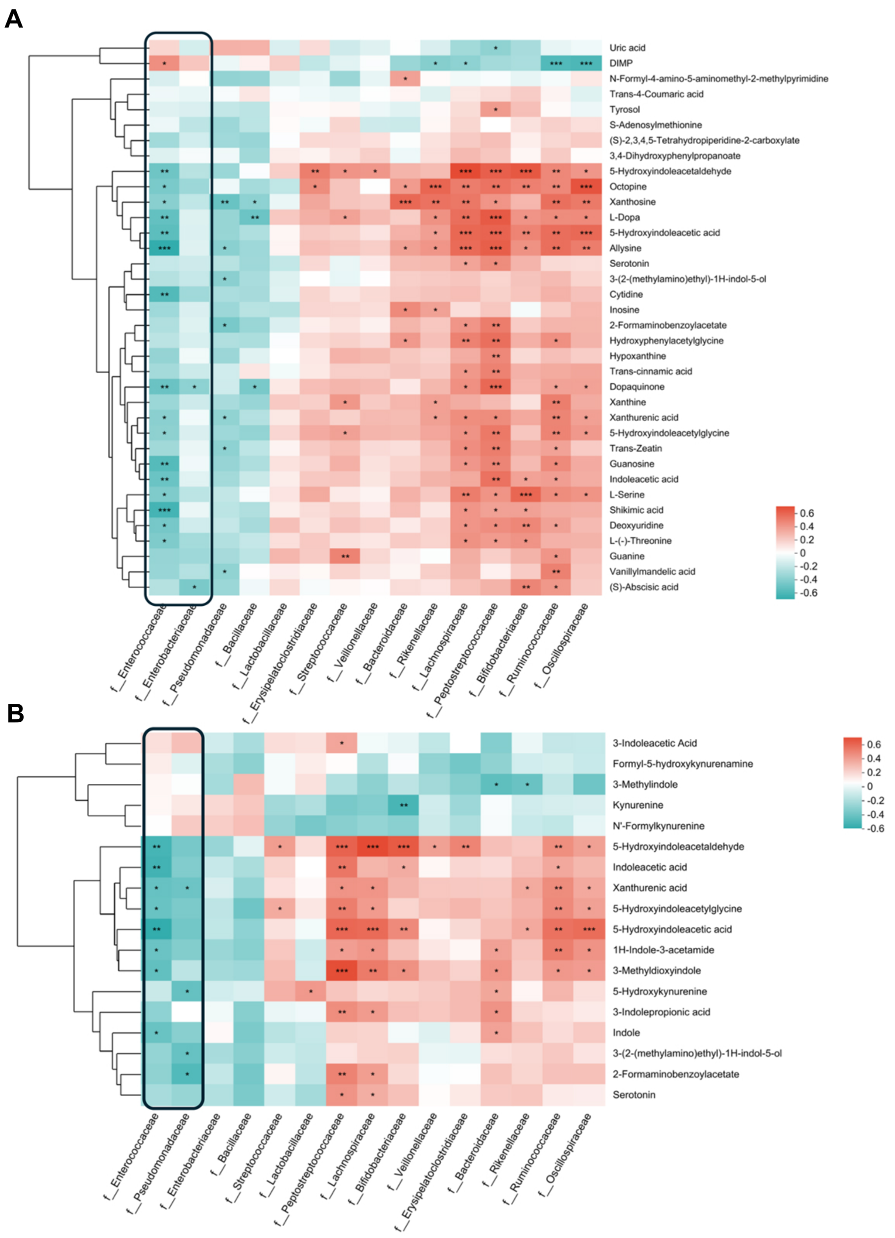

Figure S13 Correlations between gut microbiota and metabolites.

(A-B) Spearman correlation analysis was conducted to examine the relationship between the microbiota and fecal metabolome (A) as well as the annotated tryptophan metabolites (B) in a subset of AP patients (n = 32). *P<0.05; **P<0.01; ***P<0.001

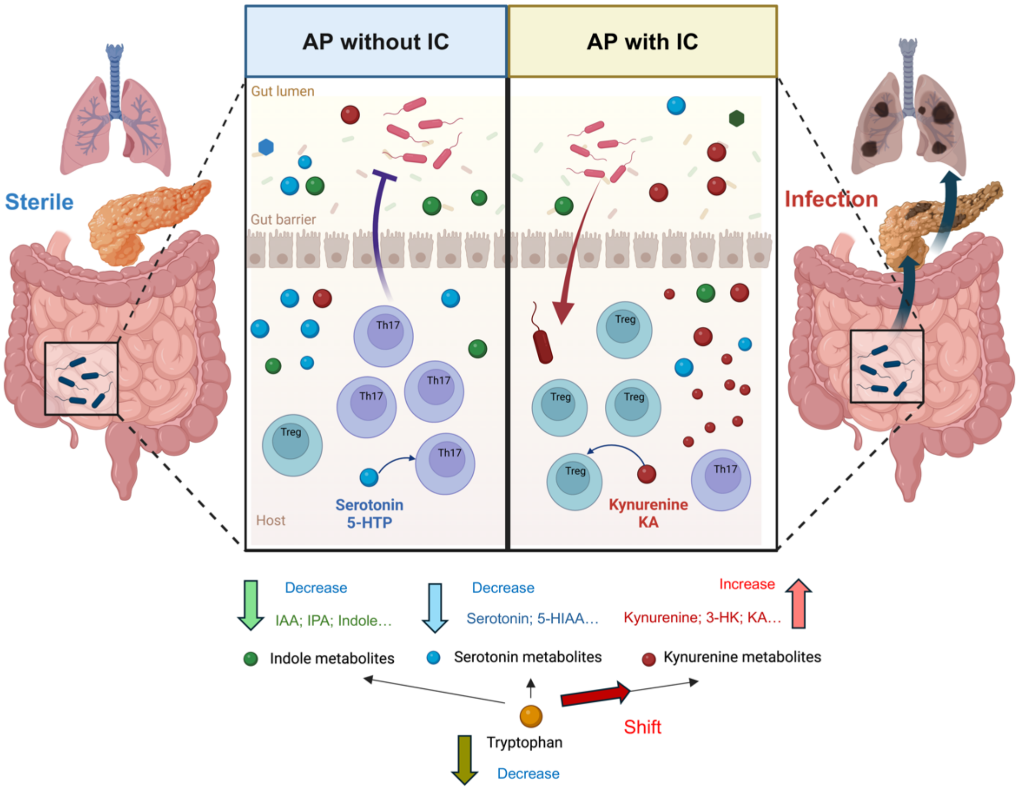

Figure S14 Schematic illustration of dysregulation of tryptophan metabolism in AP.

Intestinal bacterial translocation is considered a key contributor to infectious complications (IC) in acute pancreatitis (AP). In this study, we identified a dysregulation in tryptophan metabolism in AP, characterized by increased kynurenine metabolites (red circles), along with decreased serotonin (blue circles) and indole metabolites (green circles). This imbalance is even more pronounced in those who develop AP with complications. Additionally, our findings highlight a protective role for the Th17 response against bacterial infection in AP, modulated in opposite ways by serotonin metabolites and kynurenine metabolites, the former one can enhance Th17 response, while the last one can convert them into immunosuppressive Tregs. Here, we propose a hypothesis that this disrupted tryptophan metabolism in AP promotes bacterial translocation and subsequent infectious complications by influencing Th17 cells and their regulatory counterpart, Tregs, in distinct ways. The schematic was created using BioRender.
